# Rock art and frontier conflict in Southeast Asia: Insights from direct radiocarbon ages for the large human figures of Gua Sireh, Sarawak

**DOI:** 10.1371/journal.pone.0288902

**Published:** 2023-08-23

**Authors:** Jillian Huntley, Paul S. C. Taçon, Andrea Jalandoni, Fiona Petchey, Emilie Dotte-Sarout, Mohammad Sherman Sauffi William

**Affiliations:** 1 Griffith Centre for Social and Cultural Research, Griffith University, Gold Coast & Nathan, Queensland, Australia; 2 Australian Research Centre for Human Evolution, Griffith University, Gold Coast & Nathan, Queensland, Australia; 3 Place, Evolution and Rock Art Heritage Unit, Griffith Centre for Social & Cultural Research, Griffith University, Gold Coast, Queensland, Australia; 4 Waikato Radiocarbon Dating Laboratory, Te Aka Mātuatua—School of Science, University of Waikato, Hamilton, New Zealand; 5 School of Social Sciences, Discipline of Archaeology, University of Western Australia, Perth, Australia; 6 Sarawak Museum Department, Kuching, Sarawak, Malaysia; University of California Santa Cruz, UNITED STATES

## Abstract

Gua Sireh, located in western Sarawak (Malaysian Borneo), is known for its rock art. The cave houses hundreds of charcoal drawings depicting people, often with headdresses, knives and other accoutrements. Here, we present direct radiocarbon dates and pigment characterizations from charcoal drawings of two large (>75 cm), unique Gua Sireh human figures (anthropomorphs). To our knowledge, these are the first chronometric ages generated for Malaysian rock art, providing insights into the social contexts of art production, as well as the opportunities and challenges of dating rock art associated with the Malay/Austronesian diasporas in Southeast Asia more generally. Previous archaeological excavations revealed that people occupied Gua Sireh from around 20,000 years ago to as recently as AD 1900. The site is within Bidayuh territory, and these local Indigenous peoples recall the cave’s use as a refuge during territorial violence in the early 1800s. The age of the drawings, dated between 280 and 120 cal BP (AD 1670 to 1830), corresponds with a period of increasing conflict when the Malay elites controlling the region exacted heavy tolls on the local hill tribes. We discuss rock art production at Gua Sireh in this context of frontier conflict and Bidayuh resistance.

## Introduction

A fundamental challenge facing rock art researchers, and all those who study the human past, is understanding the age of their subject. Securely situating rock images in their chronological and cultural context is the foundation for interpreting them [1:7]. With the right circumstances for generating numeric age determinations scarce, those scientific dates that are produced become anchors for relative rock art sequences and broader insights into the human past [2]. In recent years, dating has been a major focus of Australasian research owing to revelations that the earliest phases of rock art in central Island Southeast Asia are contemporary with, and in some cases older than, the Franco Cantabrian rock art found in the deep caves of Europe; compelling evidence that disparate early human populations were independently, simultaneously producing complex symbolic imagery on cave/rockshelter surfaces [3–6].

Island Southeast Asia’s rock art spans more than 45,500 years and was produced sporadically up until the recent past [3–6].

Taking a multifaceted approach to understanding two large anthropomorphic figures at Gua Sireh, we focused on recent ‘Austronesian style’ drawings [5,7–11]. Used here, Austronesian denotes an association with the Neolithic communities who colonized Southeast Asia more than 2,500 years ago [12]. Originally a linguistic construct, ’Austronesian’ has been used by archaeologists following Peter Bellwood’s foundational model to refer to a suite of material culture thought to mark the expansion of Malayo-Polynesian Austronesian speakers (with a linguistic homeland in Taiwan) across Island Southeast Asia and the Pacific [12]. We note the utility of the term ‘Austronesian’ in relation to the Holocene rock art traditions of Island Southeast Asia and the Pacific is currently cause for reflection amongst scholars as the cultural complexity of the Neolithic period in the region continues to emerge from the archaeological record.

Using radiocarbon dating, we situate the Gua Sireh drawings in their culture-historic context, comparing the rock art with material culture excavated within the cave from the 1950s to late 1980s [13,14]. To explore human-environment interactions and past technologies, we characterized the pigments applied to draw these images. Using pigment characterizations and details of the site’s history, we discuss technical complexities relating to the age estimates [2,15–17]. Finally, we interpret these large Gua Sireh human depictions in the colonial setting in which they were made, informed by the oral histories of the Bidayuh Indigenous peoples who have continuing custodial responsibilities over the site today. This work is situated amongst recent studies across the globe that have emphasized the role of rock art in Indigenous resistance to colonial occupation, including violent frontier conflicts, even enslavement [18–23].

### Study rationale

**Fig 1:**
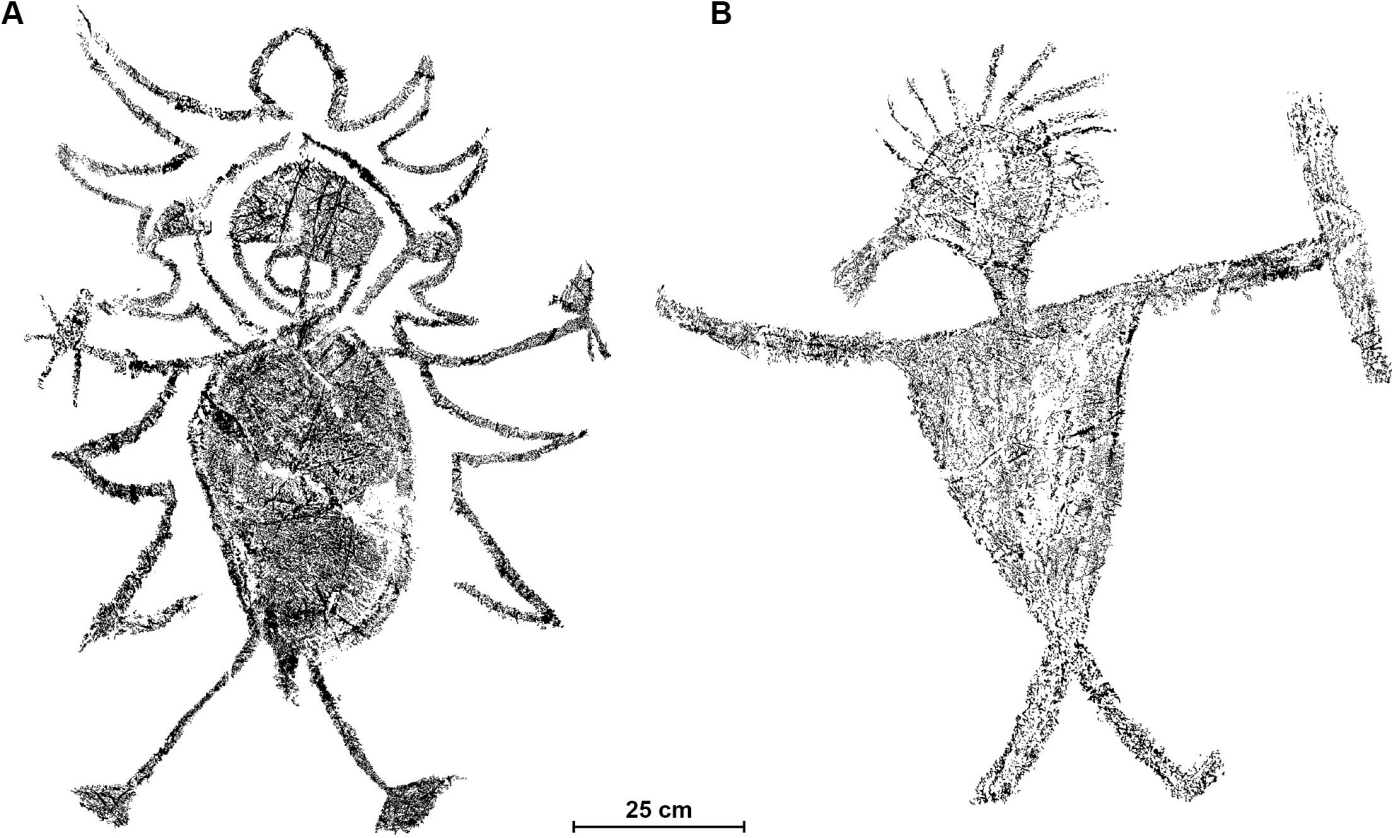
Digital tracings of the two large human figures dated in this study. A: Anthropomorph–GS3 sample taken from the left foot; B: Anthropomorph–GS4 sample taken from the left arm.

The aim of this research was to understand when the large human figures at Gua Sireh were made. We targeted the two largest motifs in the cave (Fig 1, corresponding to our samples GS3 and GS4) to test the viability of radiocarbon for dating Gua Sireh motifs, with the aim of not adversely visually impacting the rock art (see photographs before and after sampling in S1 Text). In addition, we selected samples that would aid in the interpretation of age determinations, exploring the taphonomy of the rock art panels and pigments (samples G1 and G2, details in S1 and S2 Texts). We sampled a weathered anthropomorphic figure at the entrance to the main art panels in an area of active cave surface (GS1), as well as a ‘stick figure’ motif (GS2) known from previous recordings to have been produced sometime after 1989, but before 2010. In so doing, we deliberately selected a location that we reasoned would be challenging for carbon preservation (GS1) and a recent artwork that could help address the likelihood of ‘the old charcoal problem’ (GS2) whereby an older age is returned as a result of aged charcoal being used to create a recent image [24,25]. In addition, the recent stick figure was sampled to check for potential contaminants as it was made on a panel surface thought to have been modified during active conservation management works undertaken in the 1980s (GS2) (see photographs in S2 Text).

### Background

**Fig 2:**
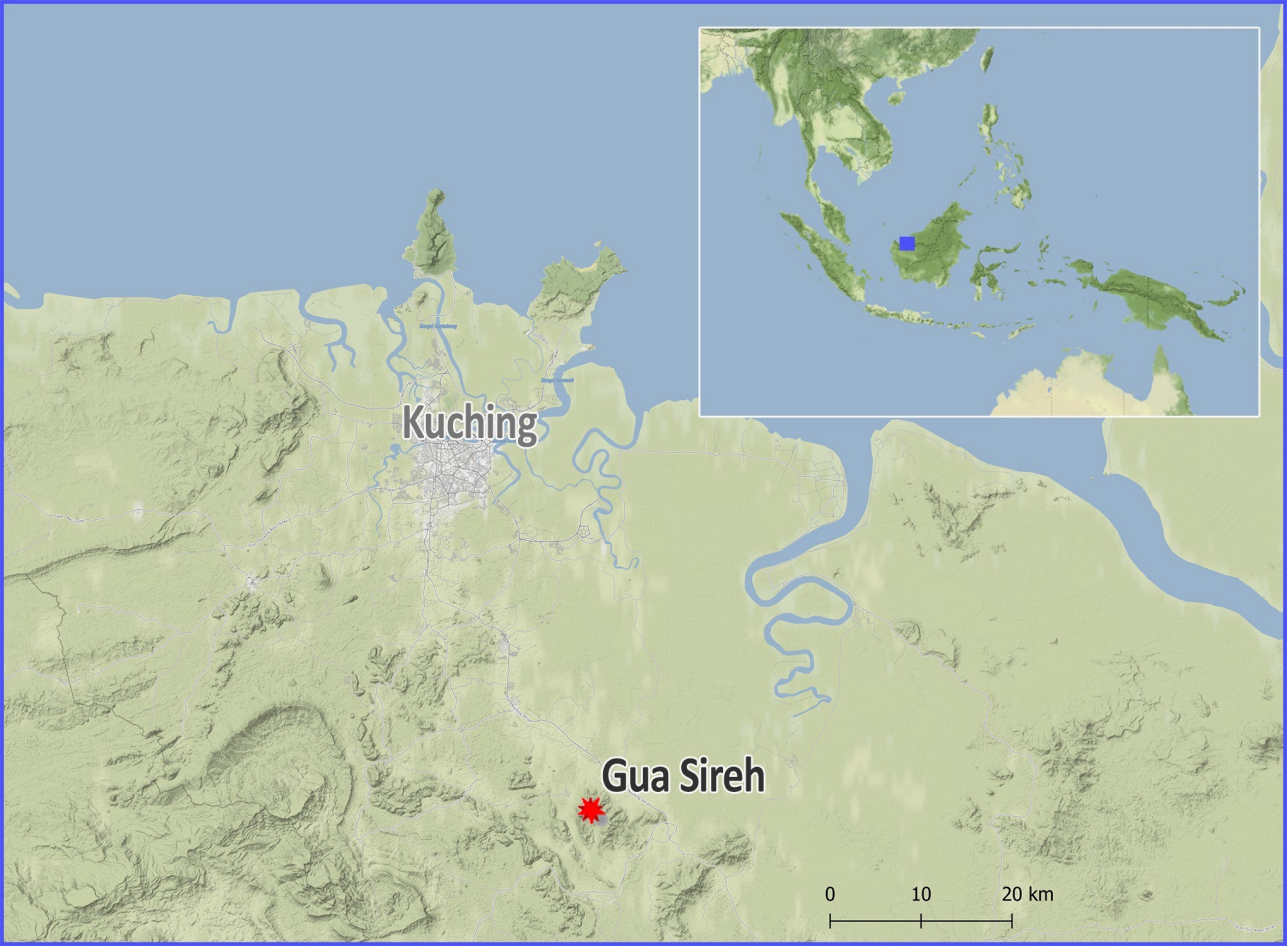
Location of Gua Sireh (Latitude 1° 10.9’ N and Longitude 110° 27.7’ E) relative to the city of Kuching (coordinates after Datan 1993) on the island of Borneo (inset).

Gua Sireh, sometimes referred to as Gua Sirih, is one of Sarawak’s best-known rock art sites. The black drawings are well-published and the site highly visited, attracting hundreds of people each year [14,26–28]. Managed by the Bidayuh (local Indigenous) community at Kampung Plaman Bantang in collaboration with The Sarawak Museum, the cave lies approximately 55 kilometers southeast of the Capital city of Kuching in a limestone hill known as Gunung Nambi [13:1] (Fig 2).

In 1959, Bidayuh elder Bidu anak Bandan (then ∼ 75 years of age) recounted the creation mythology of Gua Sireh:

“This cave at the beginning was a big ship. This ship meant to collect diamond in Gunong Sadong … about 3–4 hours walk (∼ 30 km to the northeast) from Gua Sireh now… the diamond in Gunong Sadong was kept in a large snake’s mouth. It … happened (at) the time when the main ship came to Gunong Sadong. The snake was asleep and the diamond was still in its mouth. The crews in the ship … grabbed the diamond from the snake’s mouth and sailed away with it.

Some hours later the snake awoke and found its diamond lost (gone), being raged with anger the big snake rush with all its strength to pursue the thief, at length it sighted the ship with the diamond and (struck) the ship by its tail. The lifeboat on board the ship fell and was changed to stone … called Batu Ajong … halfway between Kampong Tai and Kampong Bantang.

The ship itself after fought hard were at last turned up into a mountain called Gunong Nambi [29].

The story ends with the upturned ship transforming into a stone mountain (Gunong Nambi) and the cave being carved out by the enraged mythical snake once it reached the ship/Gunong Nambi. The creation story for nearby Gua Kapur (Kapur Cave, known today as Fairy Cave, located ∼ 30 kms to the northwest of Gua Sireh) ends similarly, but this time the protagonist is a woman angered by the actions of others, resulting in their village and all inhabitants being turned to stone, forming Gua Kapur [30:286]. Though the author was not identified in The Sarawak Museum record, it is highly likely that this account was collected by Raphael Nyandol, the ‘assistant’ of William R. Geddes, who conducted ethnographic fieldwork in the area around Gua Sireh from 10 February 1949 to 1 January 1951.These are the only two Sarawak rock art sites for which Nyandol, a fellow Bidayuh, recorded creation stories. Where creation stories for other caves without rock art in the region were recorded, the mythology is quite different [e.g. 30:284–5].

**Fig 3:**
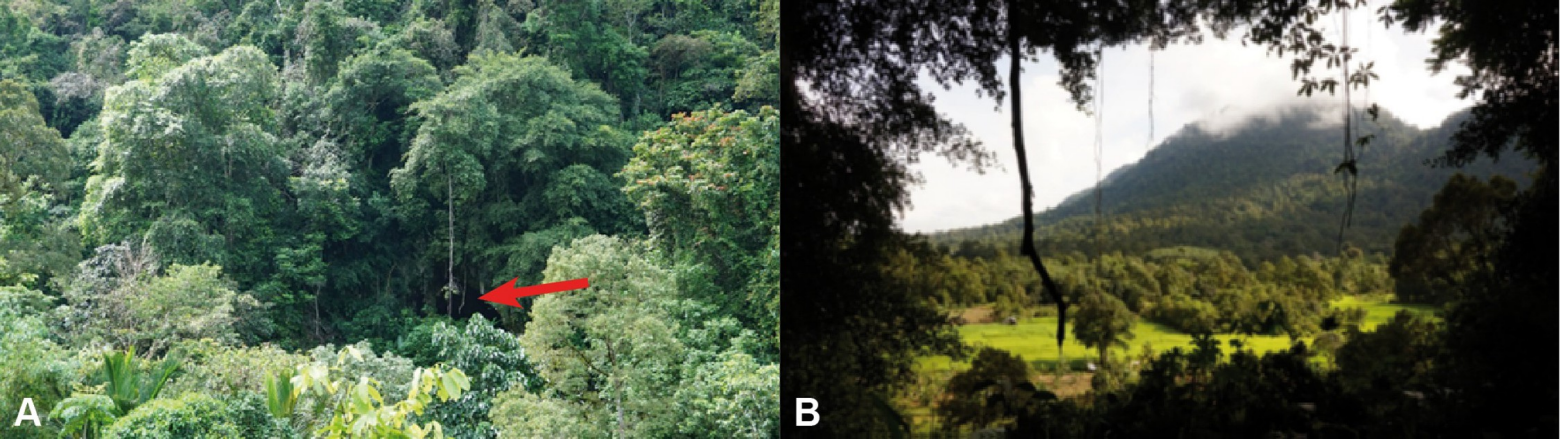
Gua Sireh main chamber entrance and outlook. A) The entrance to Gua Sireh looking south (red arrow) ∼60 meters above the valley floor; B) outlook from the cave entrance northeast down to the valley (photographs taken by Paul S.C. Taçon).

**Fig 4:**
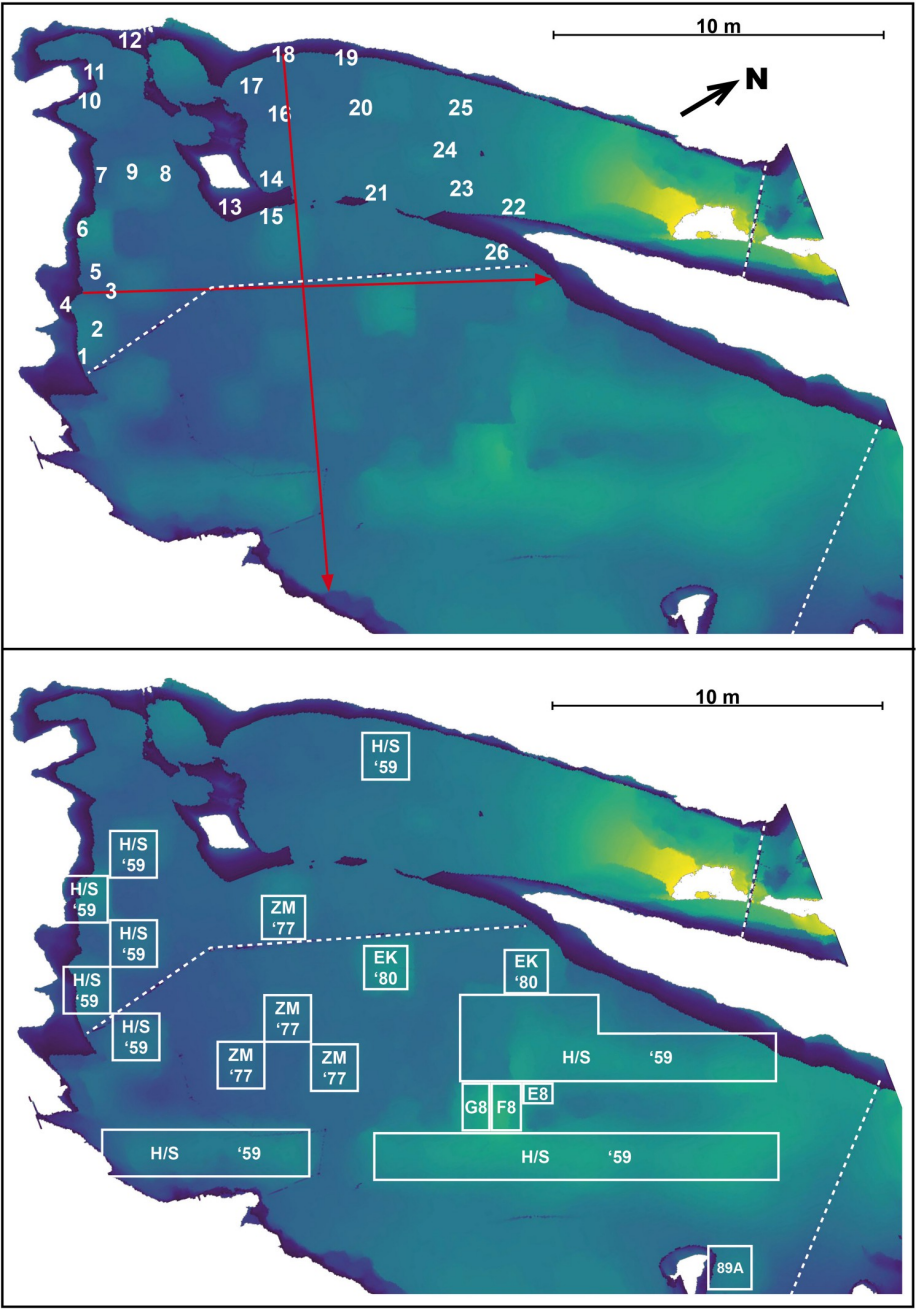
Plan view, digital elevation model of the floor of Gua Sireh. Lighter colors show a steeper gradient of topography. Dashed lines indicate the location of chain wire fencing. *Top*: Numbers show the location of panels at the site. Red arrows indicate the location of cross-sections taken from the 3D model, bisecting the drawings of large humans dated in this study (panel no. 3 relates to our sample GS3 and 18 to GS4). *Bottom*: Overlay of excavation locations adapted from [13:21]. Abbreviations for excavators and dates undertaken: H/S ‘59 = Harrisson/Solheim (1959); ZM ‘77 = Zuraina Majid 1977; EK ‘80 = Edmond Kurui 1980; E8, F8, G8 and 89A = Ipoi Datan 1989.

**Fig 5:**
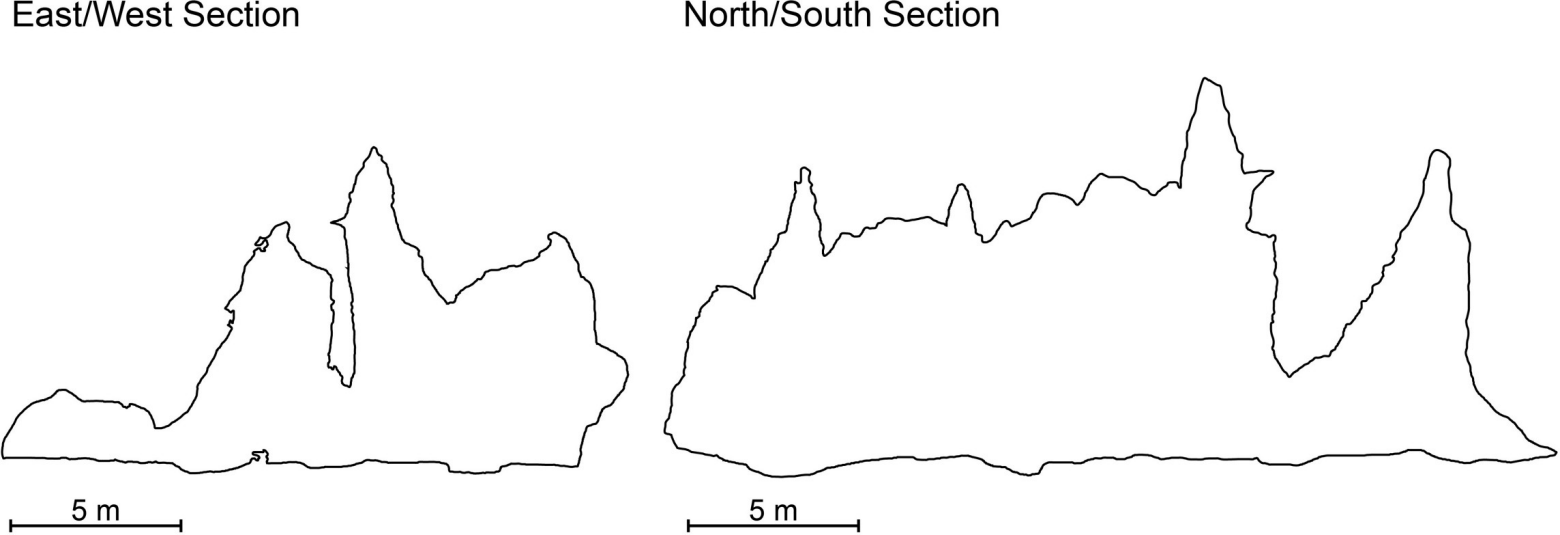
Cross sections bisecting the large anthropomorphic drawings dated in this study. *East/West Section*: Panels 3, sample GS3. *North/South Section*: Panel 18, sample GS4.

**Fig 6:**
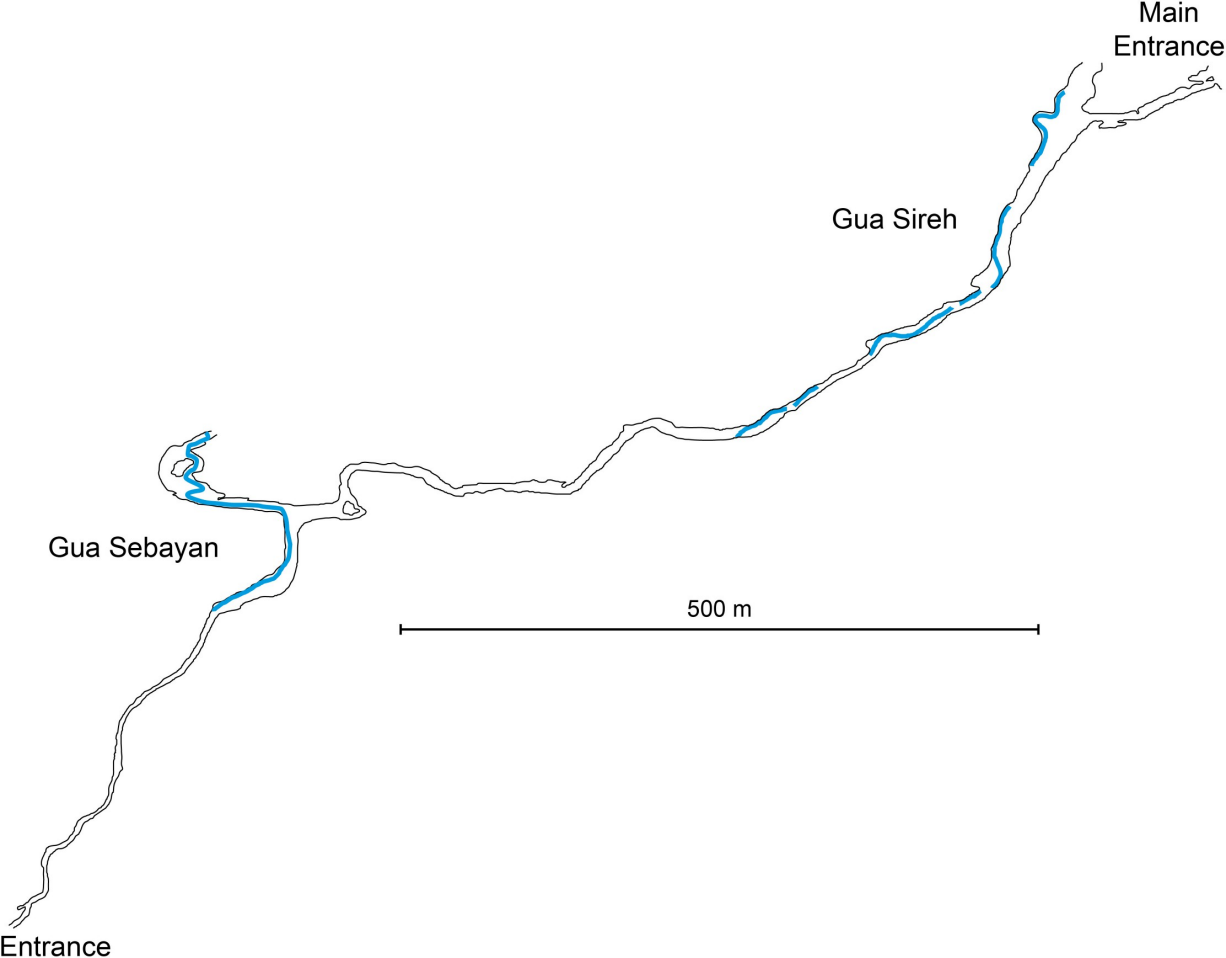
Plan overview of the Gua Sired cave system. Shows the passage through Gunung Nambi (limestone hill) and the connecting passage between Gua Sireh and Gua Sebayan. Blue indicates the location of water (including underground rivers) (after [31:101], Fig 65).

Gua Sireh has two northeast-facing entrances to the main chambers (Figs 3–6) roughly 60 meters above rice fields at the base of Gunung Nambi [13:7]. Recorded rock art is confined to these entrance chambers. The larger chamber is about 17.5 meters wide and the smaller has a width of about 4 meters (Figs 3 and 5). Beyond the entrance, at the back of the largest chamber, a passageway leads down to another large cave (Gua Sebayan), crossing an underground river, and providing access through Gunung Nambi (Fig 6).

Following smaller ‘tests’ in 1954 and 1958, the main chamber was excavated in 1959, 1977, 1980 and 1989 (Fig 4, [13,14,32]). These earlier excavations suggested that hunter-gathers intermittently occupied Gua Sireh from around 20,000 years ago [13:163, 32:150]. The Mid-Holocene record at the site yielded important insights into the introduction of rice, domesticates such as dogs and pigs, pottery with distinct manufacturing techniques and production phases over time, and polished stone adzes. Austronesian influence at Gua Sireh dates to this period between 4,200 BP and 2,000 BP, first indicated by the appearance of charred rice and pottery [13:163, 25,32:150, 33], including sherds with rice temper [34]. The deposits from ∼2,000 BP contain iron artifacts, glass beads and human burials (minimum number 16), though the bones recovered were fragmentary and fragile [13:164, 35]. Burials included grave goods such as trade beads demonstrating contact between the Bidayuh (inland tribes) and coastal traders [13:243]. Evidence for continued site use during the 1700s-1800s includes Chinese ceramics [13:163, 36:183]. Gua Sireh appears to have been abandoned ∼AD 1900 [36:183].

### Gua Sireh’s rock art

Private secretary of the Rajah of Sarawak (James Brooke) and later British Consul in Brunei, Spencer St John [26], wrote prolifically about his experiences in Sarawak. However, in his account of a visit to Gua Sireh he did not mention the rock art. Among the first non-indigenous people to note the figurative motifs were archaeologists Wilhelm Solheim and Tom Harrisson in 1959 during their excavations [14:38–39]. An initial description of Gua Sireh’s rock art was produced by Lindsay Wall [28], who classified the drawings into four categories: ‘Pinmen’; ‘Birdmen’; ‘Large’ (the subject of our investigations); and ‘Animals’. However, it was Solhiem [14:39] who provided the first published description of interacting groups of simple stick or straight-line human figures on the back wall of the cave, including a few ‘imaginary’ beings and the large ‘cloaked’ anthropomorph on panel 3 that we report age determinations for here (GS3), which he interpreted “to be supernatural in some way” [14 and Plate XI].

In 1989, Ipoi Datan [32:208–240] recorded and described over 100 charcoal drawings at Gua Sireh. More recently, between 2010 and 2015, Rachel Hoerman has taken an inventory of over 333 previously recorded motifs and documented a further 73 black drawings [7:98, 101]. Datan and Bellwood [26:103] provide a useful summary, estimating that about 36 linear meters of cave wall was used to house rock art, located in a band starting ∼1 m above floor level and then extending for ∼2 m in height. The drawings, ranging between 10 cm and 1.5 m in height, are predominantly figurative (85%), consisting mainly of anthropomorphic (human) figures with triangular bodies, some with a solid fill. A few figures were noted as wearing headdresses: some armed with shields, knives and spears. Scenes were noted as showing ‘familiar’ activities such as hunting, butchering, fishing, fighting, dancing and possibly processions or ritual ceremonies.

In 1980, as part of management works at the site, the rock art was divided into panels for documentation purposes with red encircled numbers painted on the wall to designate these groupings ([37:2]–relating to the numbers on our Fig 5). While scientific dating was yet to be undertaken directly on the motifs, Datan [26,32] proposed a maximum age for the rock art of 4,500 years based on the first appearance of pottery and other Austronesian material culture in the deposits. Considering minimum ages, Datan noted that it was possible the rock art was made after St John’s visit in 1855 as he had not mentioned the drawings, however, he “expected (the rock art) to be more than 100 years old” [13:160].

### The broader regional rock art sequence of Southeast Asia

The charcoal drawings of Gua Sireh appear to be part of the wider distribution of black drawn motifs found from the Philippines through central Island Southeast Asia across Borneo and Sulawesi to Peninsular Malaysia, thought to be associated with the Austronesian diaspora [5,7–9,11,38–40]. Black drawings have been noted in central Island Southeast Asia from as early as 1888, specifically in Liang Lumba located in Mount Mandenlla Central Kalimantan (Indonesian Borneo) [11:166]. Black is the second most common rock art pigment recorded in Southeast Asia, after red, [9,41–43]; with small anthropomorphic figures, often in active poses, dominating the rock art corpus [44].

At the coarsest level, there are at least two temporal phases of rock imagery in central Island Southeast Asia based on direct dating and superimposition studies. The first phase, executed from at least 45.5 ka until ∼ 20 ka, is only found on older cave surfaces with distinctive case-hardening (none of which were observed at Gua Sireh). This Pleistocene art is characterized by hand stencils and figurative paintings of large-bodied endemic land animals in outline profile with irregular infill, painted in red/mulberry-hued pigments. The second, more recent rock art phase corresponding to the Gua Sireh assemblage is found on fresher cave surfaces, occasionally superimposed over the case-hardened Pleistocene surfaces and art styles, and is attributed to the Neolithic communities colloquially known as ‘Austronesian’ who colonized Southeast Asia ∼4,500–2,000 years ago [13,26,33,39,40,45,46]. This younger art phase usually consists of applied black pigment (presumably charcoal) and is characterized by generally small images of anthropomorphic figures, often with triangular torsos, frequently in active poses and/or dynamic scenes, often accompanied by domestic fauna such as dogs, as well as an array of geometric, abstract and linear motifs [8,40,45].

This latter tradition of black rock drawings is found particularly across the northern half of Borneo [7:43]. A relative chronological sequence based on rock art superimposition has been proposed for the rock art of Borneo with early hand stencils, followed by multiple phases of ochre painted art and a final period of black drawings [7:45, 47:6]. Northwest Borneo’s rock art (the Malaysian states of Sabah and Sarawak) is dominated by black drawings of anthropomorphs, animals, ships and geometric and linear designs–with only a single art site containing paintings (red) found at Gua Kain Hitam in the Niah Cave complex [7:45].

Published recordings of similar black anthropomorphs with filled-in triangular torsos in central Island Southeast Asia include Gua Hagop Bilpo in Sabah [7,48], Gua Cincin in Kelantan [26,41,49–51], as well as at caves and rock shelters in Indonesia containing many hundreds of motifs including more than 30 sites in East Kalimantan (Indonesian Borneo), among them Tanjung Lokan, Kapusa Hulu and Batn Cap, Kayong [7,9,42,46], Liang Lumba in Central Kalimantan (Indonesian Borneo) [7,11:166, 48], and numerous sites across the Maros-Pangkep and Bone regencies of southern Sulawesi [8,9]. There is considerable diversity in the graphic conventions of the black drawings of the region, particularly the anthropomorphs with more slender and elongated forms noted across central Island Southeast Asia and into Peninsular Malaysia [38,39,51–53], consistent with the Austronesian drawing style developing and changing over time as observed in the black pigment rock art of the Pacific [38,43,54,55].

### Chronometrically dated black Austronesian-style rock art

Only two other chronometric age determinations from black drawn rock art motifs in the broader region have been published, both small anthropomorphs with arms outstretched upwards and angular infilled torsos, with both studies only recently completed [8,38].

A radiocarbon determination of 3570–3460 cal BP (Wk-51453) has been obtained for a (presumably charcoal) black anthropomorphic motif in Hermoso Tuliao Cave, one of the 12 rock art sites in the Penablanca Cave complex of the Philippines. The surprisingly old age for the motif is all the more remarkable considering the advancing degradation of rock art and notable motif loss within most of the complex, estimated to be as much as 62% of the pictogram assemblage evidenced by comparing rock art documented by a series of researchers from the mid-1970s to today (for details see [38:929, 931]), though Hermosa Tuliao and Segundino Tuliao caves were noted to be in good condition. The Hermoso Tuliao date challenged the notion that all black pigment rock art in Southeast Asia is recent (i.e. only hundreds of years in age) and indicates the anthropomorph was made by the ancestors of one of two current ethnic populations in the Philippines; Austronesian or Agta peoples [38:931].

At Leang Bulu Bettue in southern Sulawesi, a human figure drawn using wood charcoal on the ceiling panel was radiocarbon dated to 1580‒1430 cal BP (Wk42768). This figure is preserved on the fresher surface of the ceiling, adjacent to remnants of a presumably Pleistocene-aged, case-hardened layer retaining red pigment [8:2–3].

Previous research has suggested that, though regional variance is present, as described by Ballard [56], the distribution of the Austronesian Painting Tradition is generally limited geographically to the Banda Sea [12,41,43,57]. The assemblages from which anthropomorphic figures that have direct age determinations are available in the Philippines, southern Sulawesi and (here) Sarawak (Malaysian Borneo), occur predominantly in inland caves [7–9,37] as noted by O’Connor and colleagues for this style of rock art in Timor [12,44]. This is contra to the common sea cliff placement of rock art initially proposed as characteristic of Austronesian Painting Tradition [39,43,58, c.f. 44].

### Black pigment characterizations in Southeast Asia

In the broader Southeast Asian region, work has shown that a variety of black materials were used as rock art pigments. For example, studies in the Philippines identified rock art pigments as carbon black in Minori Cave and bone black in Segundino Tuliao and Hermoso Tuliao caves [38,59]. Though no characterisation was conducted on the pigment from the directly dated anthropomorph at Hermoso Tuliao Cave, its qualities were noted by FP as consistent with a carbon black [38:929].

Coal (mineralized carbon) was found to have been used to smear a finger line of black paint onto a 27,000-year-old trimmed slab of limestone in Leang Bulu Bettue, southern Sulawesi. Also recovered from the same deposits were as yet unidentified black (and red) pigment on a bone ‘blow pipe’ presumably used for stencilling [60:S1 27–28]. In addition, the ‘Austronesian’ style, black drawn human figure on the rock shelter ceiling was made using plant charcoal [8:3]. Natural manganese accretions have also been noted on buried limestone slabs from Leang Bulu Bettue and cave walls of rock art sites throughout the broader Maros-Pangkep regency [60: Si 27].

Finally, in Borneo (East Kalimantan), black (along with red and purple) rock art paints at Jufri Cave have been argued to be a hematite mineral pigment [42], though we note that such a characterisation could equally come from the incorporation of the case-hardened Pleistocene cave surfaces of the region which contain ubiquitous iron minerals including hematite [3,8].

### Potential radiocarbon contaminants

Complexities of radiocarbon dating are well recognized, including potential contaminants for age determinations using rock art pigments [15,16,61]. Most notable in relation to black, charcoal rock art pigments are the impacts from aged/ ‘old wood’ that has been sitting in the environment for an extended period (potentially many hundreds of years or more), or long lived trees with an inbuilt age (potentially one hundred or more years), being burned to make charcoal for pigment [17,62–64]; and/or charcoal created during much earlier burning events being used in more recent rock art production inflating age estimates by hundreds, thousands or even tens of thousands of years [25,65]. In the case of Gua Sireh’s rock art, it is unlikely that fallen timber would survive for long in the tropical climate and jungle environment surrounding the cave, making the use of aged wood less likely. Furthermore, wood charcoal prepared for rock art production is considered less likely to use large trunks that could retain inbuilt ages, and more likely to use smaller diameter species and/or branches, especially for drawing.

The management history of Gua Sireh is also pertinent, as the site has been actively conserved for more than 50 years. There were a series of measures implemented in 1980, including physical interventions where: “Wire brushes and various chemicals were used to remove … profanations and a preservative of polyvinyl acetate in toluene was sprayed on the drawings” [14:Pl. XIIIb, 38]–see S2 Text for further details]. To maintain the ‘natural appearance’ of the wall, museum staff and contractors re-covered cleaned areas by dusting them with material from the floor of the cave [37:1]. Thus, there is potential for both old and modern carbon to have been incorporated onto the black drawings of panels 6 and 7 as a result of management work. We, therefore, avoided sampling rock art in this location (except GS2, which we knew from recordings was recent).

Finally, sample GS1 was taken from a motif in an active part of the cave wall surfaces where calcium carbonates, evaporate mineral such as gypsum, microbial activity and resulting carbon mineral deposition such as oxalates may have been present [2:12]. While pre-treatments applied would have negated any impacts on the resulting age determination this is a moot point as the sample did not return enough carbon to generate a target.

## Materials and methods

### Digital recording

During fieldwork in August 2019, Gua Sireh was recorded in 3D by AJ using photogrammetry and laser scanning. ∼ 1,000 photographs were collected using a Canon 6D camera and 35 mm lens and then processed in Agisoft Metashape v. 1.5.1. A portable Leica BLK360 terrestrial laser scanner (TLS) was used to capture 32 scans of the cave, focusing on the areas with rock art. As the accurate point cloud output of the TLS often lacks the color definition necessary for rock art research, TLS scans and photogrammetry images were combined in Reality Capture 1.1.1 to create a photo-realistic model [66,67]. Unfortunately, not all photographs were able to be integrated into the 3D model. The location of Panel 26 is an estimate, while the locations of all other panels are accurately plotted on the sitemap (Fig 4). Finally, orthomosaics were also produced to assist the detailed figure analysis (see S3 Text).

### Scanning electron microscopy

SEM analyses were undertaken by JH at the Queensland University of Technology Central Analytic Facility using a field emission scanning electron microscopy (JSM-7100F) to image surface morphology. The spatial distribution of chemistry was investigated using electron dispersive X-ray spectroscopy (a JED-2300F EDX) in both spot assay and element-mapping modes. Samples were placed directly on metal stubs with adhesive carbon tape and platinum-coated for conductivity. Secondary Electron images were collected at 5Kev. EDX analysis was undertaken using the backscatter detector at 20Kev. EDS undertook analysis and interpretation of the resulting micrographs (S4 Text). SEM investigations took place prior to ^14^C dating of the pigments, to inform the pretreatment methods and the interpretation of results.

### Radiocarbon dating

**Fig 7:**
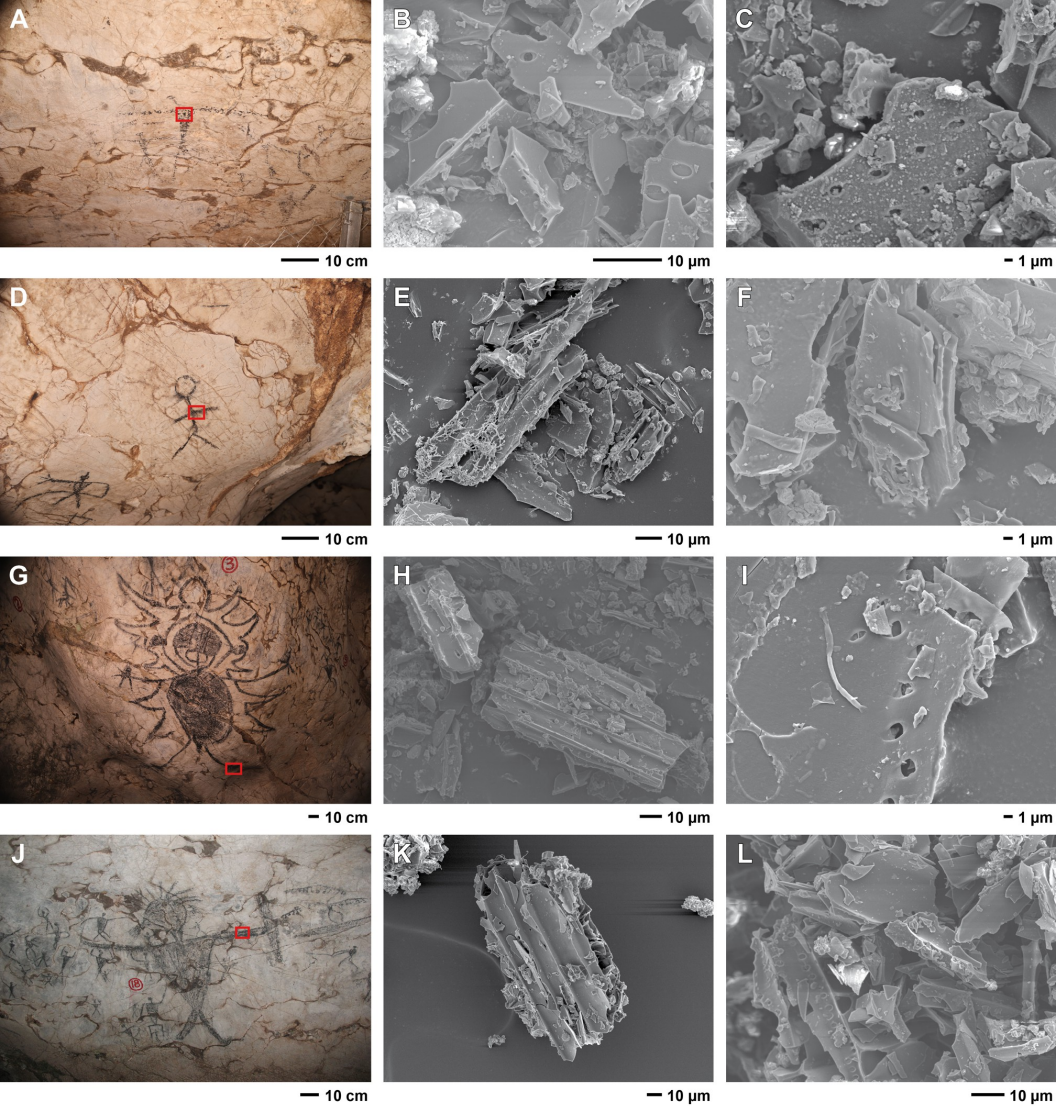
Sample location and pigment morphology. Location of dating samples (red rectangles) with inset SEM micrographs of pigment morphology. A to C: GS1; D to F: GS2; G to I: GS3; and J to L: GS4.

Four separate charcoal drawings were sampled by JH and AJ using a Dremel drill fitted with a separate dental burr for each sample to avoid contamination. GS1 was taken from a small anthropomorph at the entrance to the larger main chamber north of panel 26, above the chain mesh fence (top of Fig 4). The upper torso of this anthropomorph was selected as it appeared to have the densest pigment. Pigment from this figure was thinner than the other samples and was well bonded to the cave wall, consistent with the active calcium carbonate redeposition observed on the northern end of this panel location (see S1 Text). Consequently, GS1 had the smallest sample volume as we did not want to risk damage to the visual amenity of the motif. GS2 was taken from the body and inner left arm of a recent ‘stick figure’ made between 1990 and 2010 at the edge of panel 7. GS3 was harvested from the left foot of a large, round-bodied male anthropomorph on panel 3 whose embellishments include facial features, (presumably) clothing, and him holding knives. GS4 was taken from the upper left arm of the large anthropomorph with a triangular body, holding a sheathed knife on panel 18 (sample locations within the various motifs are illustrated in Fig 7 below, while S1 Text contains detailed photographs before and after sampling). With the deliberate exception of GS1, we chose motifs at Gua Sireh that we felt confident had not been affected by the application of hydrocarbon-based products applied during previous conservation management works undertaken in the rock art panels in 1980 (S2 Text).

Samples were processed at the Waikato Radiocarbon Dating Laboratory in New Zealand. All samples were washed in hot 10% HCl, rinsed and dried to remove contaminants. Carbon-13 stable isotope values (δ^13^C) were measured on prepared graphite using an AMS spectrometer. The radiocarbon dates have therefore been corrected for isotopic fractionation. Dates were calibrated using Bronk Ramsey 2020 (OxCal v4.4.2) and atmospheric data from Reimer et al. ([68]; Northern Hemisphere calibration curve, see S5 Text).

## Results

### Radiocarbon dating

No result was returned for GS1 as the sample was too small to process reliably. The GS2 stick figure drawn sometime between 1990 and 2010 on panel 7 returned an age determination of 149 + 20 BP (170–130 cal BP or AD 1780–1820) (Wk51698). GS3, the large, embellished, round-bodied anthropomorph on panel 3 was dated to 259 + 19 BP (280–240 cal BP or AD 1670–1710) (Wk51699 R). GS4, the large anthropomorph with the infilled triangular body holding an object we interpret as a sheathed knife on panel 18 returned an age of 137 + 19 BP (160–120 cal BP or AD 1800–1830) (Wk51700 R). In summary, the most recent stick figure motif known to have been made between 1990 and 2010 returned a chronometric age for the material from which it was produced, i.e. between AD 1780–1820. The large triangle torsoed anthropomorph GS4 in the central part of the smaller chamber returned an age determination of AD 1790–1830. The oldest motif, GS3, dated to AD 1670–1710, was the most embellished in the study and the anthropomorph noted as having the most accruements of all the depictions at Gua Sireh (having facial features, wearing a distinctive cloak including a hood/headdress, and holding objects interpreted as knifes in each hand, Fig 1). GS3 is visible from the entrance of the main chamber and was therefore present when the other motifs in this study were made (see S5 Text).

### Pigment analysis

The importance of pigment characterizations for understanding and interpreting numeric age determinations of rock art continues to be demonstrated by numerous studies across the globe [69–71]. The characterized pigments used to draw four Gua Sireh anthropomorphs, even the recent stick figure, are largely uniform, indicating that the same black pigment material has been used over time. The fine structure of the carbon-rich grains, with holes at the edge of fin protrusions, indicates wood charcoal [72–75] (Fig 7). EDX analysis confirmed the elemental composition of the large plate morphology grains are almost exclusively carbon, consistent with charcoal (details in S4 Text). The sparse organic microstructures observed on the surfaces of grains resemble bacteria/microbial mats (spherical and worm-like structures, and the long strands with spherical structures on their ends–visible in panels B and L of Fig 7, though see S4 Text for more detail). This agrees with rock art panel taphonomy observed elsewhere in Island Southeast Asia [3,8,60].

The sparse nature of microorganics, and the lack of case hardening on the panel surfaces, supports the assumption Gua Sireh’s broader rock art assemblage is likely hundreds of years in age, and may be at most thousands (rather than tens of thousands of years), as no Pleistocene rock surfaces appear to survive in the cave. It is also clear that there are geological salts and other evaporites on the rock art surfaces and that a small amount of the limestone upon which the rock art is executed was incorporated during pigment sampling. Again, geological salts and other evaporites on the rock art surfaces are consistent with panel taphonomy in the region [4,8,42], and the relatively sparse build-up of geological salts (presumably gypsum) or other evaporates indicates remodelling of the limestone surfaces for hundreds or, at most, thousands of years.

Sample GS4 was observed as the most highly fragmented charcoal with incorporated environmental detritus/rock matrix particles (panels K and L Fig 7). However, the charcoal morphology remains consistent with the other samples and the cell structures that can be observed are directly similar to those of GS3 in particular (Fig 7 and S4 Text). Therefore, we consider the fragmentation more likely reflects a different pigment preparation and or application in this case, rather than different composition (i.e. this is wood charcoal, not bone black, but it has been ground finer before application to the rockface, or has been rubbed with more repetitions to draw the motif).

Taxonomic identification of plant species used to produce the charcoal pigments was attempted by EDS using the SEM images produced by JH. Identifiable cell structures and anatomical features were compared with currently available Indo-Pacific anthracological reference collections/descriptions [see 76,77]. GS1 and GS2 both presented highly fragmented cell structures. GS1 included some clearly visible, small to minute bordered pits (Fig S4.3 in S4 Text) and simple pits, sometimes alternate, consistent with xylem vascular tissue and potential fibres from hardwood tree taxa. The presence of both alternate simple and bordered pits potentially indicates the use of two different wood taxa for the GS1 pigment.

The GS2 sample, though highly disintegrated, preserved some cell structures ‐ small simple alternate pits consistent with xylem vascular tissue (Figs 7E and S4.7 top and centre in S4 Text). Hardly any other anatomical elements were observed so no taxonomical identification can be proposed, other than vascular plant charcoal.

The GS3 and GS4 samples presented similar cell structures with relatively better integrity, showing grouped fibres with bordered pits (Figs S4.10 top centre, S4.11 and S4.15 bottom left and bottom right *in S4 Text*) that could correspond to elements of vascular bundles, characteristics of monocotyledon stem. GS4 also presented a larger element with opposite simple pits coherent with xylem vessel (Fig S4.16 top left *in S4 Text*).

Given the fragmentary nature of the vascular tissue observed, taxonomic identification remains difficult at a higher level for GS3 and GS4. However, the clear presence of grouped fibres, associated to a xylem vessel (albeit in isolation) indicates the pigment used for these drawings could come from a monocotyledon stem, rather than a hardwood species. The opposite simple pits observed in the vascular element of GS4 (Fig S4.16 top left in S4 Text) correspond in particular to anatomical features observed in tropical bamboo species, as described for *Gigantocloa*, *Bambusa* and *Dendrocalamus* genera [78,79] and observed by EDS in a reference specimen of *Dendrocalamus giganteus* (Fig S4.19 in S4 Text). There are more than 10 genera and dozens of species of bamboos in the Malay archipelago, which also hosts some of the largest bamboo forests in the world, long recognised as an essential resource and cultural item for human groups in the region, including for charcoal production [80–82]. On this basis we propose that the plant taxa used for the charcoal pigment of GS3 and GS4 is a vascular plant most likely to be Monocotyledon cf. *Bambusinae*, including species of the three genera cited above. Of note is the fact that Datan [13:7] observed that the slope under the cave mouth was covered in regrowth forest including bamboo.

## Discussion

### Reliability of the age determinations

We have confidence in the radiocarbon determinations returned for the two large anthromorphs at Gua Sireh for several reasons. Firstly, we recognize the ‘old charcoal’ problem needs to be accounted for, whereby charcoal from older cultural events is used in the production of rock art [16,25,66,71], especially given GS1, taken from a motif known to have been drawn between 1990 and 2010, returned an age of 149 + 20 BP. As can be seen in the elevation model presented at the top of Fig 4, the cave floor slumps where previous excavations have been backfilled at Gua Sireh, and there is a visible change in the color of surface sediments indicating bioturbation from backfilling events. Critically, the excavations that turned the sediment to leave ‘old charcoal’ on the surface at Gua Sireh occurred from 1959 onwards, at least 150 years after the GS4 panel 18 drawing was made and more than 250 years after the GS3 panel 3 anthropomorph. Furthermore, while GS1 and GS4 returned similar ages, the appearance of the motifs on the panels are very different (see S1 and S2 Texts for detailed photographs). For instance, the GS1 stick figure has a dense pigment, with deep color saturation and crisp appearance, whereas the large anthropomorph GS4 is dull, with more dispirit pigment coverage and evidence that environmental products such as calcium carbonate and evaporite minerals have been deposited on the motif’s surface (consistent with SEM observations, see S4 Text).

While it is possible that old charcoal was used to make the large Gua Sireh anthropomorphs, or any other art at the site for that matter, it is considered unlikely that exposed charcoal would last on the surface in the tropics [38]. Similarly, the aged/‘old wood’ effect [62], where charcoal derived from plant materials that have been preserved in the environment for an extended period prior to being burned, is considered unlikely because dead wood degrades quickly in the tropics [38]. The bamabo species used to create GS3 and GS4 are unlikely to generate any significant inherited ages as they are not long-lived species (have general life spans of <100 years) and we consider that smaller diameter shoots would have been selected for making pigments. The probability of recent rock art being made using older charcoal could be further tested using the recently recorded rock art closest to the entrance of the main chamber, away from the previous excavations (i.e. away from potential old charcoal sitting on the cave floor) [7] and on different panels than those included in this study.

Conversely, there was a possibility that modern carbon could have artificially reduced age determination at Gua Sireh as a result of the hydrocarbon products sprayed on some of the drawings in 1980 when The Sarawak Museum undertook some major physical works at Gua Sireh. Graffiti was removed and “a preservative of polyvinyl acetate in toluene was sprayed on the drawings” [14:39]. According to Gasing [37:2], the polyvinyl acetate was dissolved in toluene at 3% concentration. Such contamination was thought most likely to have impacted the stick figure made between 1990 and 2010, which subsequently returned a much older radiocarbon age than it should (170–130 cal BP), most likely because charcoal from the floor of the shelter, which was bioturbated from backfill of previous excavations, was used. This is in keeping with what Bulbeck [36] reported for the upper layers of the Gua Sireh deposit after analyzing tradeware sherds recovered from Gua Sireh: “From consideration of the precise dates preferred for the sherds, the top five centimeters were deposited during the 19^th^ century, whereas the deposits between five and 15 cm depth pertain to the 18^th^ century, extending at most to the early 19^th^ century” [36:183]. Further evidence that the pigment used to draw the recent stick figure may have been opportunistically obtained from the floor comes from our characterisation studies that showed that while broadly similar with the other samples, the pigment from GS2 was distinct and could not be identified to taxa. Whilst the pigments from the large human figures preserved more morphological detail and are likely both bamboo species, the more highly fragmented nature of the younger GS4 sample compared to the GS3 anthropomorph suggests a different pigment preparation/application technique showing technological change over time between motifs produced in the late 17^th^ and early 19^th^ centuries. Similar technological divergence was seen in the GS1 sample that did not return an age determination, but showed different charcoal preparation again, potentially using multiple species of wood.

Finally, the social context of the time periods to which the two large Gua Sireh figures date, as well as further investigation into the material culture they are depicted holding are congruent with the age determinations obtained and support our interpretation of the large human figures relating to a period of frontier conflict.

### Sarawak from the 17^th^ and 19^th^ centuries and Gua Sireh as a refuge

From the 16^th^ century, Sarawak was under the influence of the Bruneian empire (i.e. ruled by the Brunei Sultanate). By the late 17^th^ to early 19^th^ centuries, when the two large anthropomorphs were made at Gua Sireh, between AD 1670–1710 and AD 1790–1830, Brunei had delegated authority across the northwest coast of Borneo to Malay elites who held influence inland along large river systems [83:36–37]. In 1858 Frederick Boyle [84:292] noted:

“All of the Sea Tribes [Iban] … were under the nominal control of Malay Governors, equally with the Hill people [Bidayuh]; (the Iban) being … much stronger race in numbers … were very apt to bully their masters. Still, they always paid them a certain revenue, though, on occasion, they retook the money by a general fine on the Malay population”.

In 1841 the Sultan of Brunei installed James Brooke as the governor of Sarawak (initially the Sarawak River area) as a reward for helping quash a local rebellion and restoring Bruneian control, thus bringing Sarawak under British influence. In 1842, the Sultan of Brunei ceded sovereignty of Sarawak to Brooke, who ruled as Raj until his death in 1868 [85].

Thus, when the older anthropomorph (GS3, panel 3) was drawn between AD 1670 and AD 1710, the Bidayuh were dominated by Malay elites, whereas the second large anthropomorph (GS4, panel 18) would have been made between AD 1790 and AD 1830 during a period of increasing conflict between Bidayuh and both Iban (Indigenous peoples from the coast also known as Sea Dayaks) and Brunei Malay rulers. During this period many Indigenous Sarawakians moved into the upland interior, including the Gua Sireh area, to escape persecution [86:42–3].

Prior to the Brooke dynasty “The Brunei rulers not only bullied and enslaved the people but also had no compunction in allowing expeditions of the Ibans to attack the Land Dayak areas (Land Dayak is a gloss for the inland Indigenous peoples of Sarawak, or hill tribes, including the Bidayuh). The Ibans kept the heads of the people they slaughtered and handed over the slaves they captured to the Brunei authority as their share of the loot” [87:247–8]. In 1840, James Brooke was told by the chief of the Bidayuh “The Rajah (from Brunei) takes from us whatever he wants, at whatever price he pleases, and the Pengirans take whatever they can get for no price at all” [88:57].

When Spencer St. John [27:223–299] entered Gua Sireh in 1855 he was shown a human skeleton by some ‘Land Dayaks’ (presumably Bidayuh) accompanying him. A ‘Sarawak man’ with him told St. John the skeleton was of a Dayak he shot “many years ago” [27:225] before James Brooke arrived in 1839, and that it resulted from a very harsh Malay chief named Bandhar Kasim:

“… he was a very harsh man, and oppressed the Dayak more than was usually the case among the neighbouring chiefs. One tribe on the right-hand branch of the Sadong had suffered very severely from his exactions. They only murmured when he took their goods; when he demanded their children they refused to give them up, and flying to the Sirih caves threw up a barricade across the entrance. This example he thought might prove contagious among the neighboring tribes, so he determined to attack them; besides he was delighted with the opportunity of acquiring slaves, as every one he captured would be reduced to that state” [27:225].

In response to this resistance, the Malay chief assembled “a force of 300 men, many with firearms”. When they began to attack, they were repelled by the Bidayuh throwing heavy stones down onto the group, rolling rocks from their elevated position in Gua Sireh [27:225]. As noted above, the cave mouth is around ∼60 m above the valley floor, dropping away to a steep rocky slope that runs down to the Bantang Kiri stream, flowing into the Bukar river [13:7]. Though they retreated, when the chief promised to give a slave to anyone who could drive the Bidayuh out of Gua Sireh ‘the Sarawak man’ volunteered. He climbed above the cave and then shot the first person he saw and swung down into the cave. In the end, two Bidayuh were shot, seven taken prisoner, with the rest escaping out through the far side of the cave complex [27:226].

The above account testifies that the Indigenous peoples living near Gua Sireh would retreat to the cave as a strategic, defensible refuge, further indication that the Gua Sireh large human figures relate to a period of turmoil and frontier conflict for the Biduyah and other displaced Dayaks. There are many such instances of rock art being produced in times of increasing stress and tension [49,89–92], as well as much recent research on contact period rock art, including studies in peninsular Malaysia [52,92].

### The large human figures of Gua Sireh as evidence for frontier conflict

While trying to avoid ‘gaze and guess’ approaches to rock art interpretation [93], the geophysical and socio-political contexts of the largest anthropomorphs at Gua Sireh bare consideration. We acknowledge the selection of motifs for this pilot dating study at Gua Sireh was primarily motivated out of pragmatic concern not to negatively affect the visual amenity of the sampled motifs; nevertheless, the age determinations provide a temporal anchor for their social context of production (outlined above). Though separated by as much as 160 years in age, these large anthropomorphs share many characteristics: namely size, relative embellishment (GS3 a cloak/headdress and GS4 a headdress), and both are holding objects we interpret as weaponry (presumed to be knives/a sheath). The rock art at Gua Sireh was made inside a large, defensible cave with readily fortified entrances, as well as access to a passage through Gunung Nambi that provides an escape route. Changes in rock art created in areas where the physical landscape afforded refugia (to where people retreated) during colonial occupations and territorial violence have been noted by many scholars [18–22,89,94,95] as have depictions of weaponry under similar colonial circumstances [9,23,39,96,97].

The large anthropomorph on panel 18 (GS4) is shown holding a large rectangular object noted in previous recordings to resemble a traditional Borneo *Parang Ilang* sheath (sword case) [see 98:159–60]. The *Parang* was used by the Bidayuh, other Land Dayaks and Iban (Sea Dayak) as a general all-purpose blade, equally for agricultural tasks and headhunting. However, we note Parang is the Malay and Iban word for these weapons, which generally have a concave blade and concave sheath morphology [99,100:219]. We also note that during his analyses, Datan [13:151] suggested the object could also be a shield.

The shape and scale of the object held by the large figure of panel 18 is consistent with *Pandat* recorded as the war sword of the Land Dayaks, including the Bidayuh. The function of these swords is recorded as exclusively for fighting/protection, i.e. “it is never used in agriculture or handicrafts” [99:37, 100:226]. The sheaths of *Pandats* are characteristically straight, cut square at the terminations, fairly uniform in width (i.e. not tapered at the distal end like other Sarawak ‘swords’) (ibid), and are ∼60 cm in length and ∼10 cm wide [100:220], as opposed to the Dayak shields of the late 18^th^ and 19^th^ century which are ∼100–120 long and ∼30–40 cm wide, as well as usually tapered or rounded at their ends [101].

In regard to the much shorter objects held in each hand by the large anthropomorph GS3 on panel 3, the *Parang Ilang* was used by the Iban of Sarawak, though it is said to have originated from the Kayans, an inland tribal group Indigenous to what is now north-western Kalimantan in the center of Borneo, southeast of Gua Sireh [100]. Their morphology tended to be a shorted knife-style weapon, rather than a longer sword, with prominent, often elaborately carved, wooden hilts (see S6 Text for examples). An important record in The Sarawak Museum Accessions Books notes the Sadong Bidayuh attached *buko*, a small, unsheathed knife to their garments [99]:16, Sarawak Museum Accessions book number 717]–presumably worn tucked into belts or waistbands. It seems likely, therefore, that the large Gua Sireh panel 3 figure (GS3) is holding local blades used by both Land and Sea Dayaks across Borneo in the 17^th^ and 18^th^ centuries. The *Parang Ilang* is recorded as the principal weapon of offense used in the internecine warfare that marked the first decades of white rule in Borneo (the Brooke dynasty) [102,103:2].

Apart from the weapons depicted with the large Gua Sireh anthropomorphs, a further indication of conflict may be found in the active scene involving each figure (photographs in S3 Text). The older large anthropomorph (GS3) on panel 3 has a round body and is more embellished than most other anthropomorphs at the site with a large circular head, a mouth indicated and a defined penis, hands, and feet. He also has an unusual outline with jagged edges around the body, five triangular shapes on either side of the body, and a rounded top above the head, giving the impression that it is wearing a cloak and/or headdress (Fig 1). Five small anthropomorphs were drawn above and to the left of the large panel 3 figure’s head. Two of the smaller anthropomorphs stand on the large anthropomorph’s shoulders. Similarly, the large anthropomorph (GS4) on panel 18 has 7 small anthropomorphs around it, reaching to its body, with three that could be interpreted as standing on the figure’s shoulders. The larger size of the dated anthropomorphs and the other small human figures on their shoulders may suggest the large anthropomorphs were depictions of big and/or powerful warriors.

Doubtless, Gua Sireh housed many social groups and performed many different functions over the millennia of human occupation at the site. Indeed, the rock art has been reported as depicting a variety of activities from hunting, butchery and fishing, to fighting, dancing and possibly processions or ritualistic ceremonies [14:39, 26:103]. The direct dates produced in this pilot study prove that episodic periods of rock art production at the site can be identified. Indeed, given the ubiquity of black drawn motifs across the Island Southeast Asian region, and their probable links to the migrations of Austronesian and Malay peoples, the success of our pilot dating programs opens exciting possibilities for understanding the complexities of rock art production in the later Holocene.

Future dating programs at Gua Sireh may unpack some of the different functions and temporal frameworks of episodes of drawing over the past few hundred years. With some of the previously undocumented drawings located by Hoerman [7] at Gua Sireh and nearby Gua Bumo II having been made in the 20^th^ Century, given subject matter such as a swastika and an airplane at Gua Bumo II [7:124] and possible depictions of human figures holding firearms in a recently discovered Gua Sireh panel to the left of the entrance and far from the rest of the drawings [7:100 and 225; PT personal observation], there is also further scope to interrogate the use of old charcoal from floor deposits at Sarawak rock art sites.

## Conclusions

The radiocarbon age determinations reported here sit neatly alongside other recently published numeric ages for the distinctive black drawings associated with the migration of Austronesian people across Southeast Asia [3,5,7–11,26,38]. The Gua Sireh large human figures were produced between AD 1670 to AD 1830 at a time of frontier conflict, amid frequent territorial violence endured by Bidayuh and other displaced Land Dayaks. In the context of determinations for other black anthropomorphs in the same rock art style, drawn in the same charcoal media, which date to as early as 3,500 years ago in Hermoso Tuliao of the Penablanca Cave complex in the Philippines [38] and 1,500 years ago at Leang Bulu Bettue in Southern Sulawesi [8], the Gua Sireh human figures show that this rock art tradition continued to be produced up to 150 years ago. The coherence of style elements and subject matter (dominant human figures) across the time breadth of the black-drawn classic ‘Austronesian’ style rock art indicates that similar graphic conventions, as well as dry applied charcoal pigments, have been used to record human experiences across Southeast Asia for the past three and a half thousand years. The considerable diversity in the black drawn assemblages, particularly anthropomorphs, suggest they record the complexity of Holocene human migration and cultural interactions in the region. The success of three of the four radiocarbon targets we analyzed in this pilot investigation highlights the utility of ^14^C dating for constraining the ages of black-drawn rock art across Southeast Asia. In conjunction with detailed recording work and regional rock art analyses taking place [7,9–11,39,40,52,104], additional radiocarbon dating of black drawn rock art is poised to provide further insights into the Austronesian/Malay Diasporas, as well as the complexity of human history at Gua Sireh and broader Southeast Asia.

## Supporting information

S1 Text Photographs before and after samples were taken for ^14^C dating. (DOCX)

S2 Text Photographs of previous conservation interventions. (DOCX)

S3 Text ‘Fly through’ 3D model data links, and composite images of the dated anthropomorphs. (DOCX)

S4 Text Scanning electron microscope analyses. (DOCX)

S5 Text Radiocarbon dating summary. (DOCX)

S6 Text Example Pandat and Parang weapons from Sarawak and Sabah. (DOCX)
