# Supplementary material for: Rock art and frontier conflict in Southeast Asia: Insights from direct radiocarbon ages for the large human figures of Gua Sireh, Sarawak
Source: PLoS One. 2023 Aug 23;18(8):e0288902. doi: 10.1371/journal.pone.0288902 (PMC10446206; doi:10.1371/journal.pone.0288902)
Supplement: S1 Text — (DOCX) [file pone.0288902.s001.docx]

# Supporting Information

S1 Text: **Photographs before and after samples were taken for ^14^C dating.**

Photographs taken by Paul S.C. Taçon, 2019.

**S1** **GS1**

**
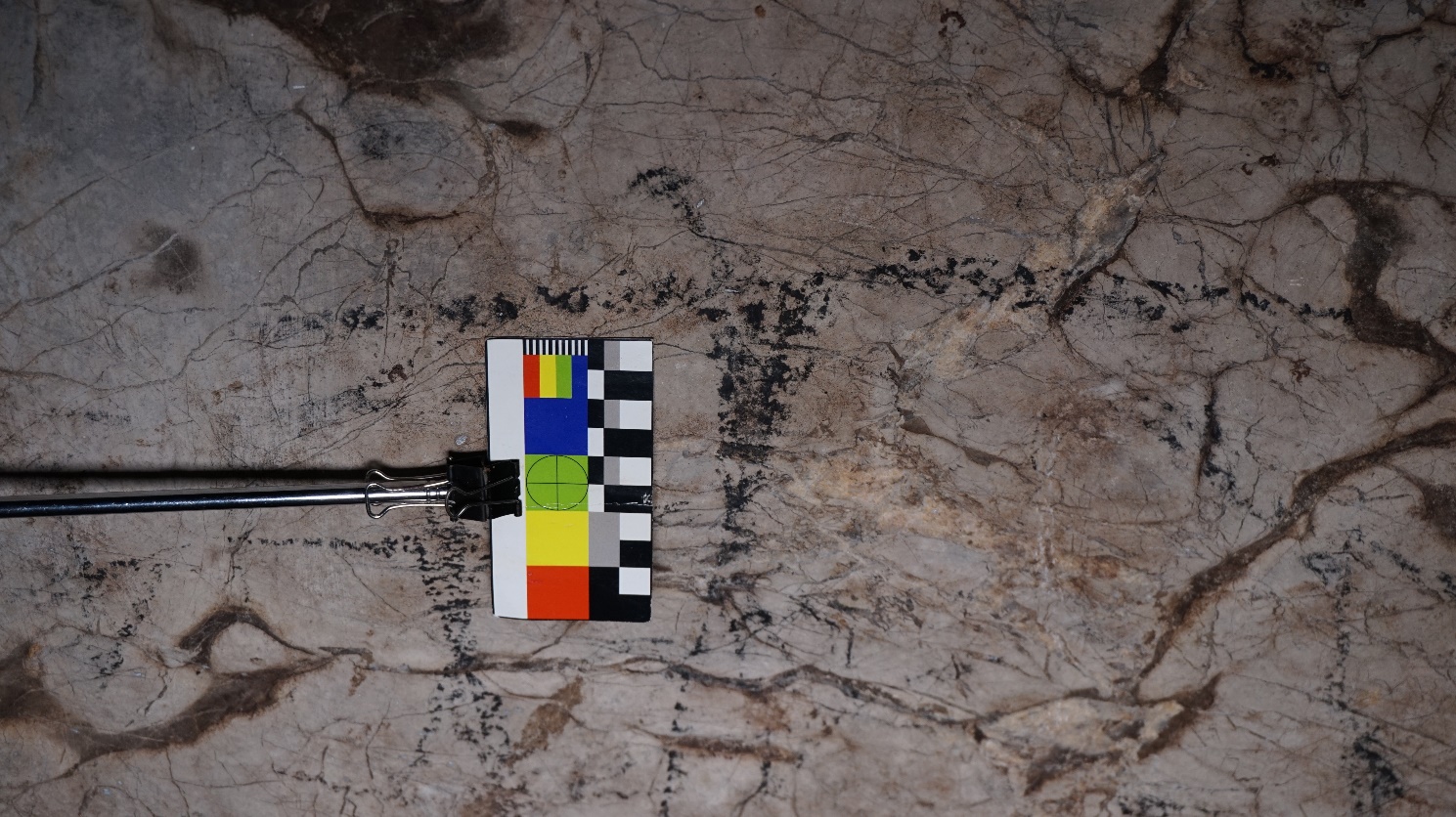
**

**GS1 Before**

**Fig S1.1.** Photograph of locale GS1 before sampling. Panel 26, Figure 4.

**
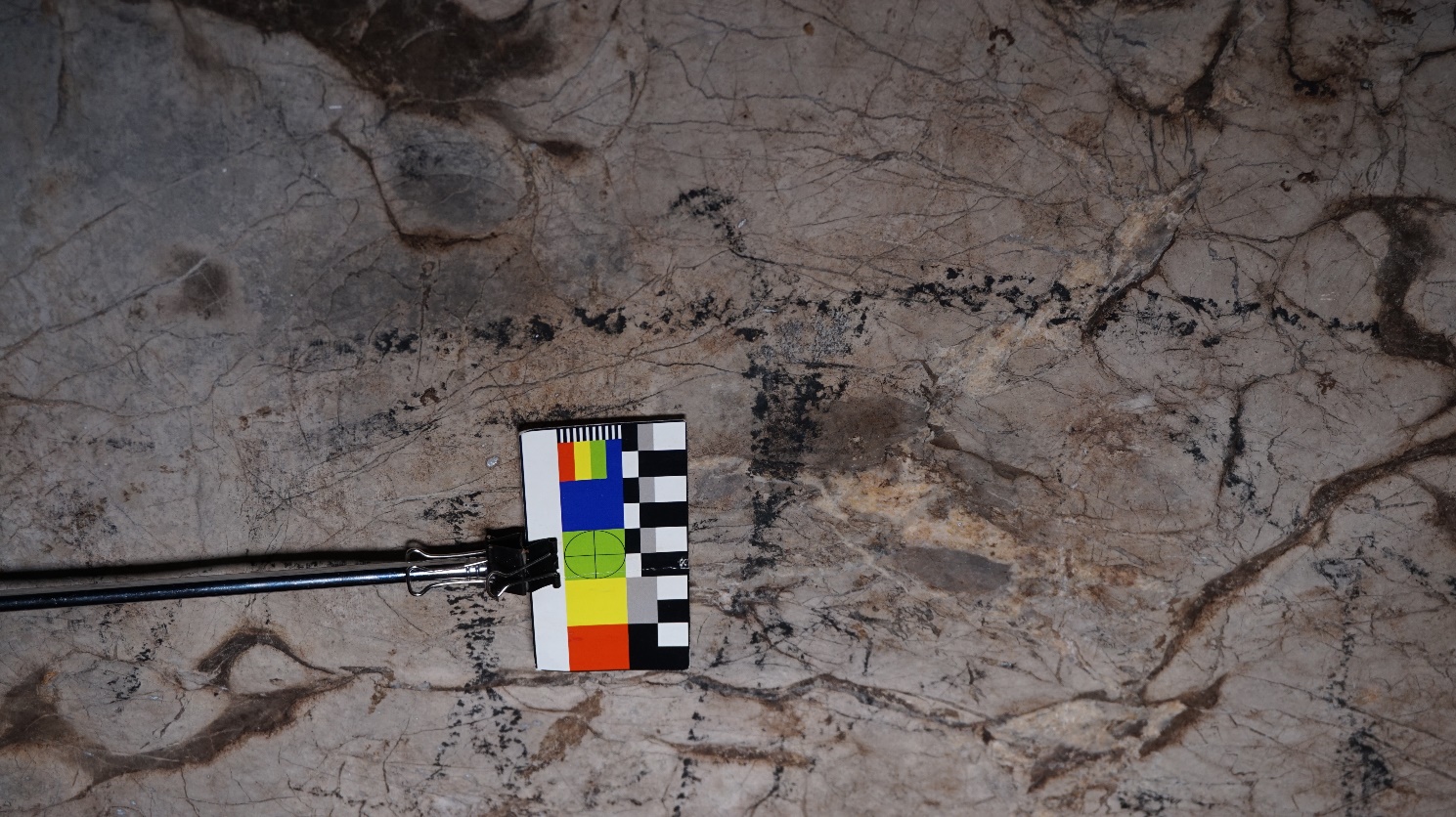
**

**GS1 After**

**Figure S1.2.** Photograph of locale GS1 after sampling.

**S1 GS2**


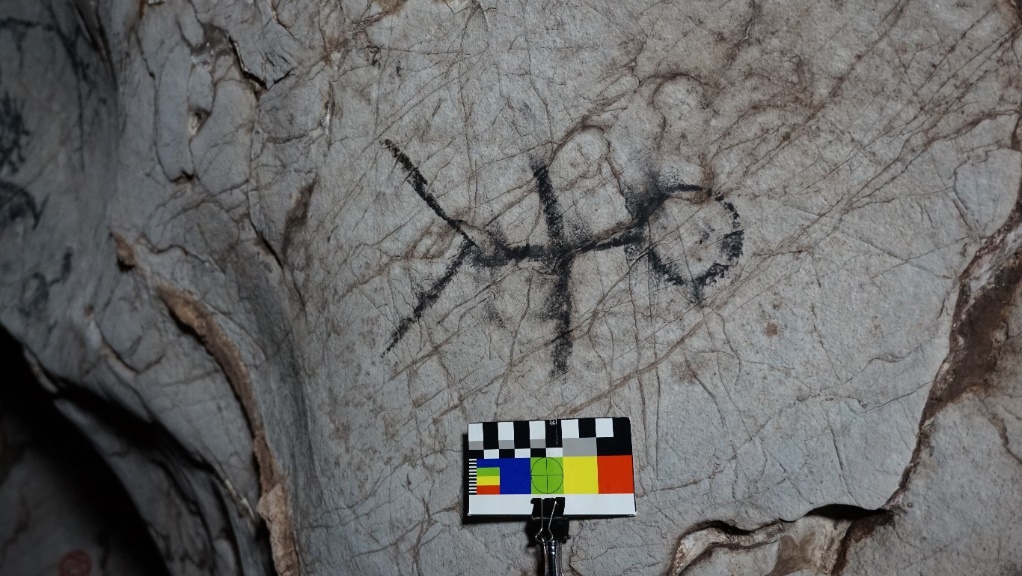

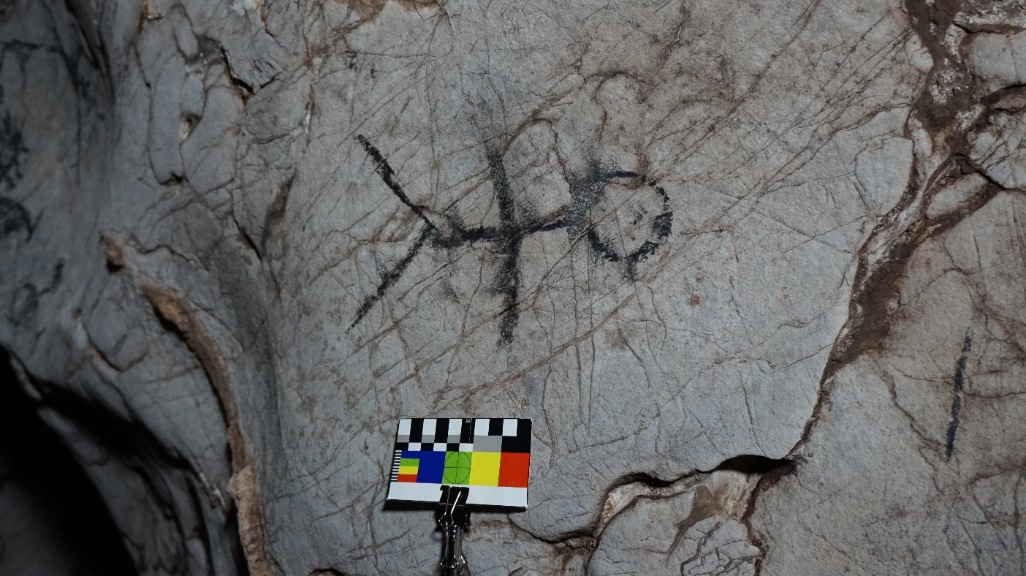


**GS2 Before**

**GS2 After**

**Figure S1. 3.** (Left) Photograph of locale GS2 before sampling.

(Right) Photograph of locale GS2 after sampling.

**S1 GS3**

**
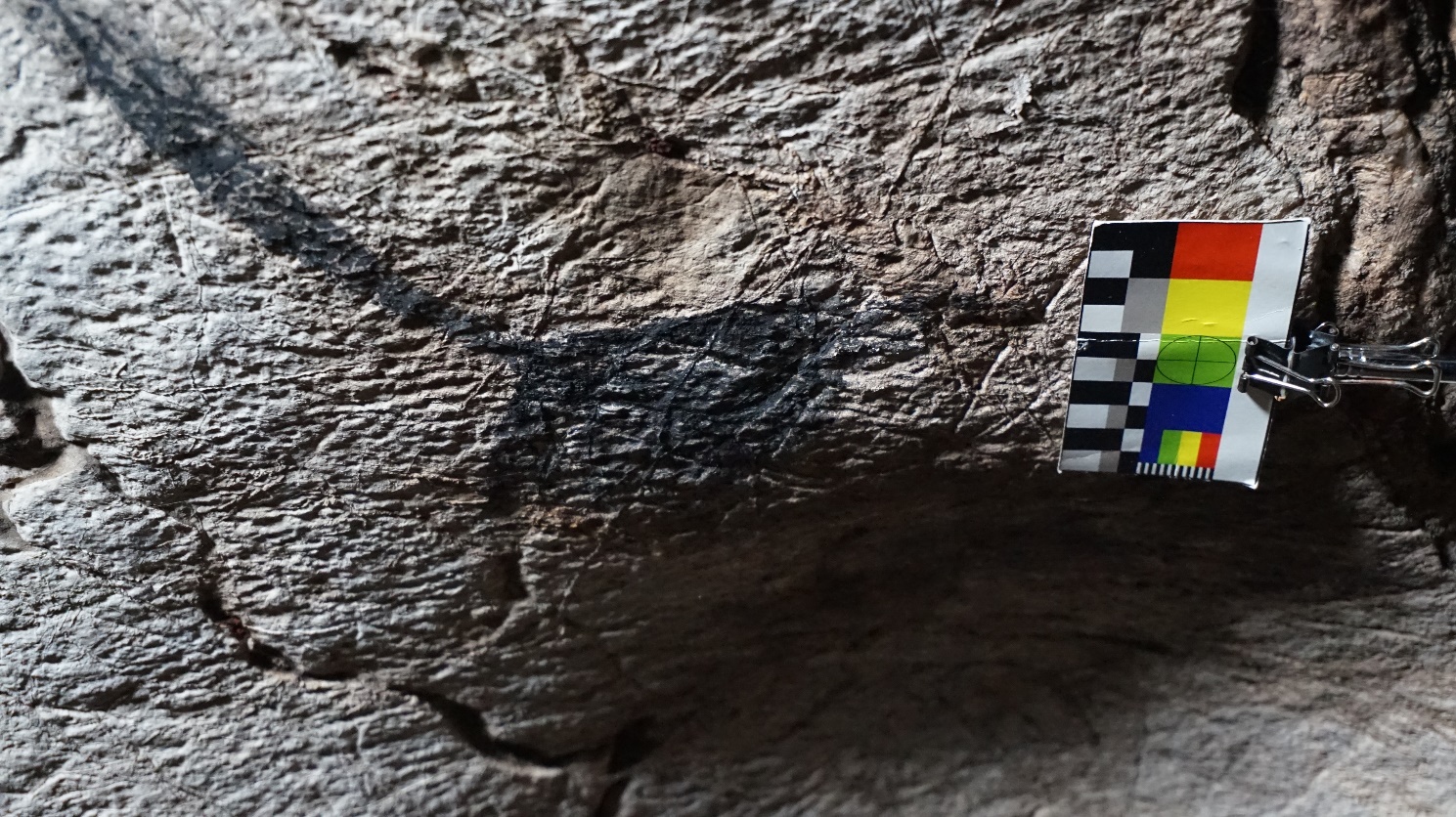
**

**GS3 Before**

**Figure S1.4.** Photograph of locale GS3 before sampling.

**
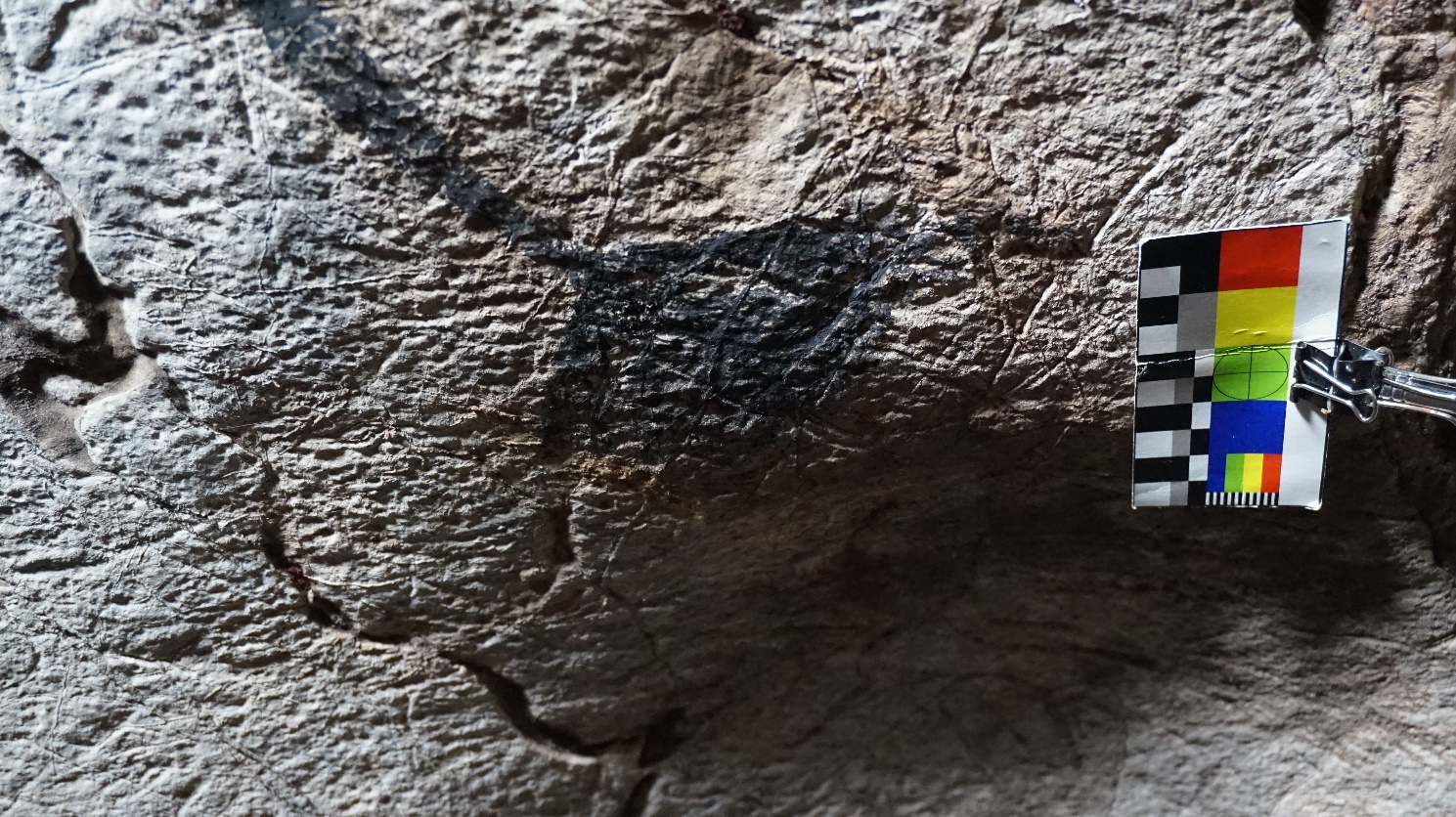
**

**GS3 After**

**Figure S1.5.** Photograph of locale GS3 after sampling.

**S1 GS4**

**GS4 Before**

**
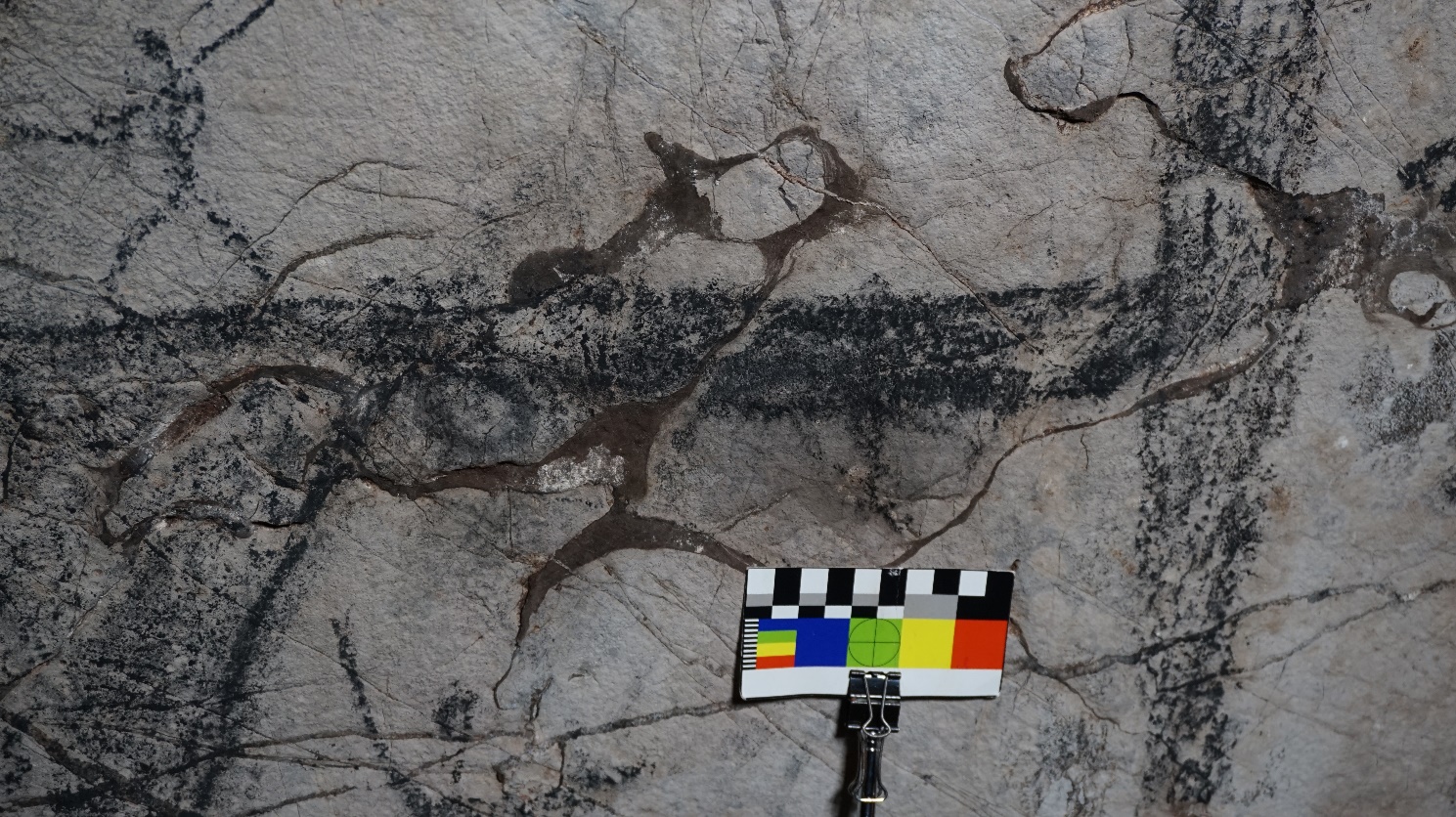
Figure S1.6.** Photograph of locale GS4 before sampling.

**
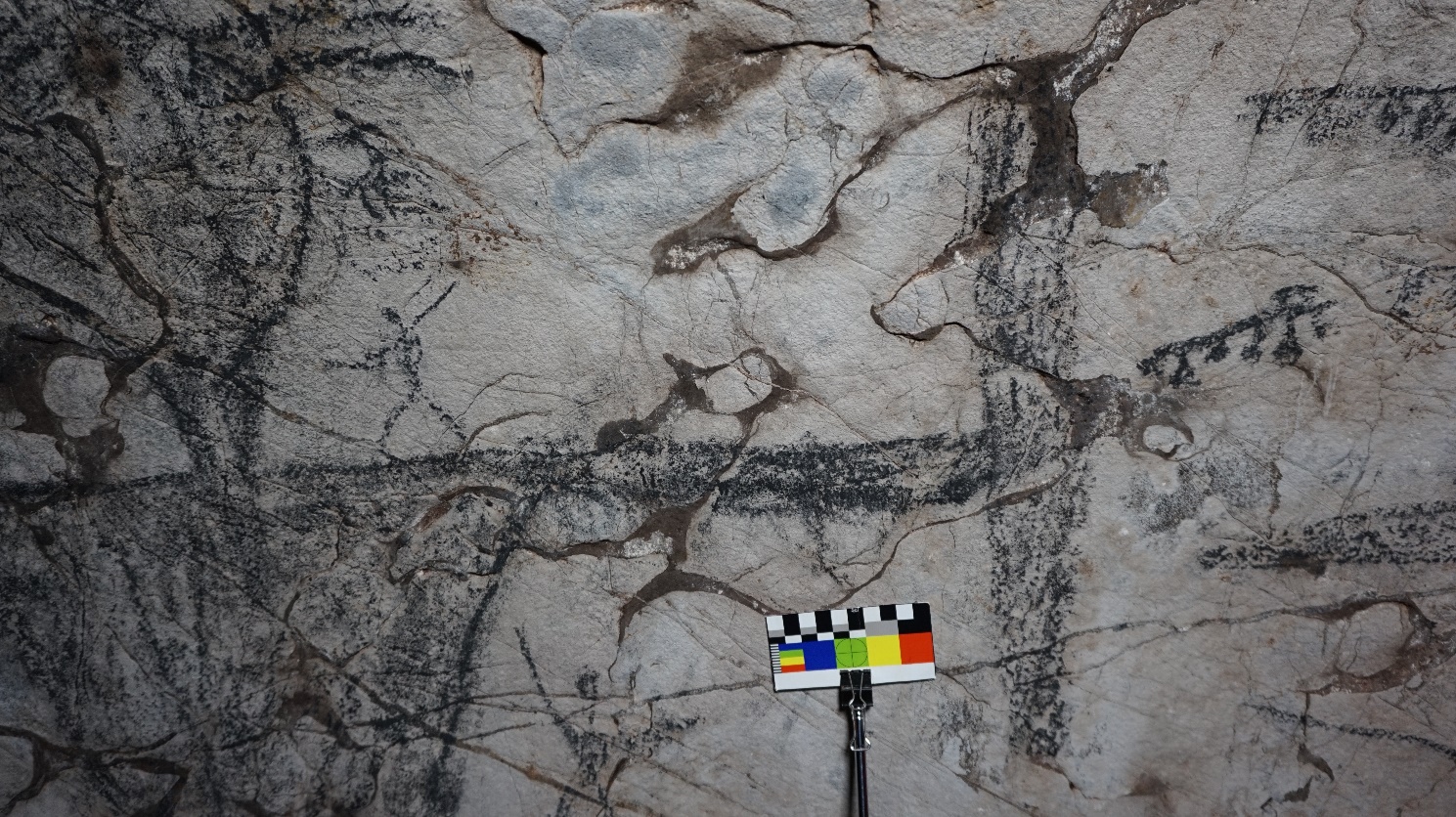
Figure S1.7.** Photograph of locale GS4 after sampling.

**GS4 After**
