# Supplementary material for: Rock art and frontier conflict in Southeast Asia: Insights from direct radiocarbon ages for the large human figures of Gua Sireh, Sarawak
Source: PLoS One. 2023 Aug 23;18(8):e0288902. doi: 10.1371/journal.pone.0288902 (PMC10446206; doi:10.1371/journal.pone.0288902)
Supplement: S2 Text — (DOCX) [file pone.0288902.s002.docx]

# Supporting Information

S2 Text: Photographs of previous conservation interventions.


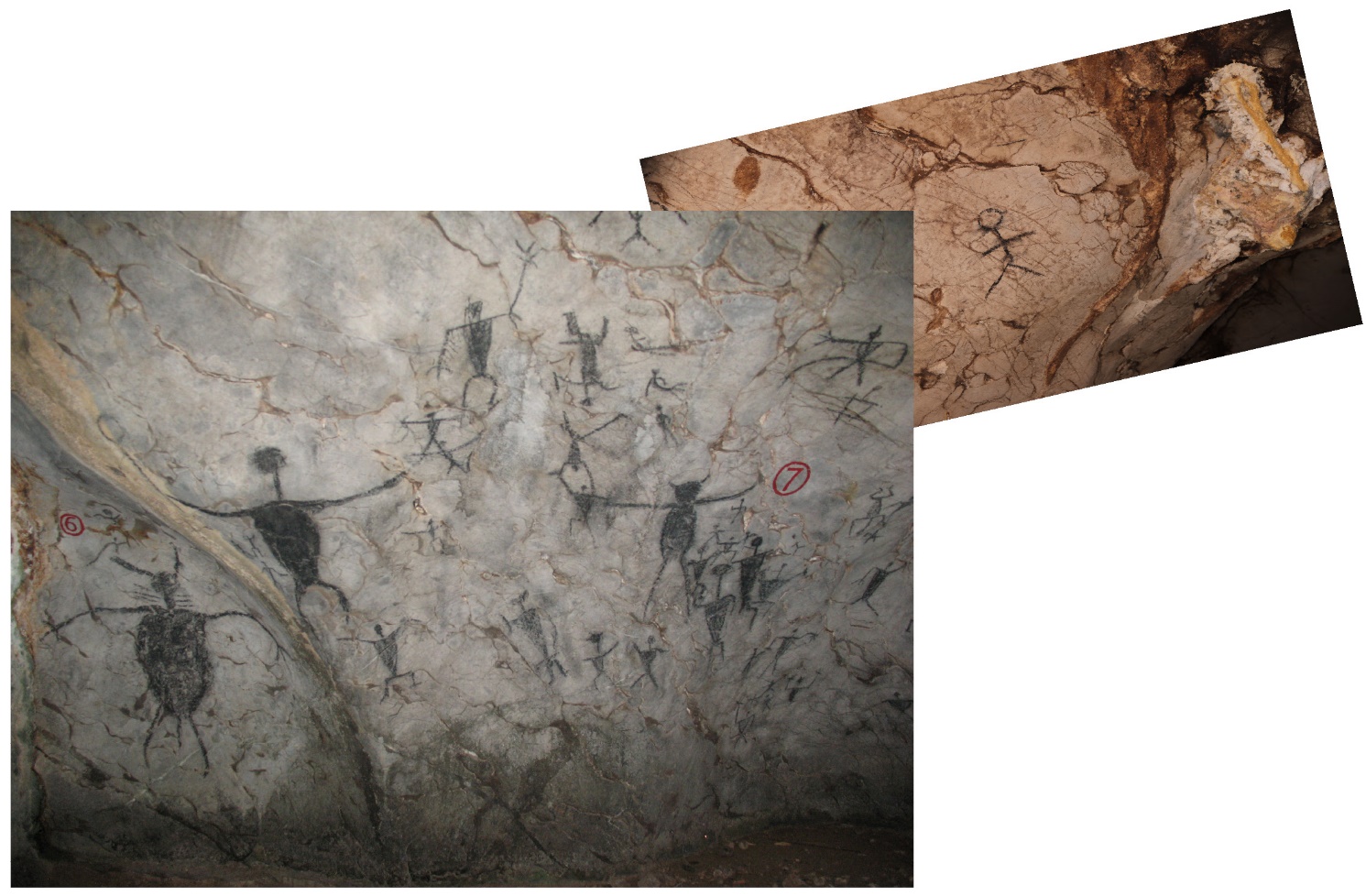


**Fig S2.1**. **Overview of Panels 6 and 7.** Photo overlay with the red arrow indicates the location of the ‘stick figure’ sample GS2 shown in the detail inset on the right (left photograph Paul S.C. Taçon 2019, right photograph Andrea Jalondoni 2019).


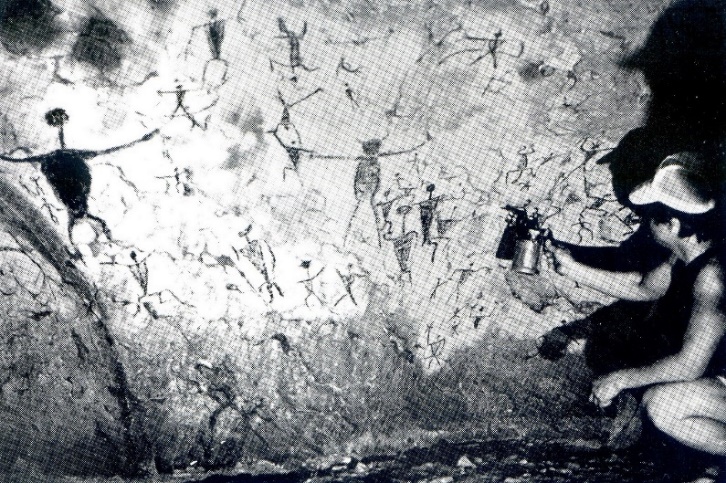

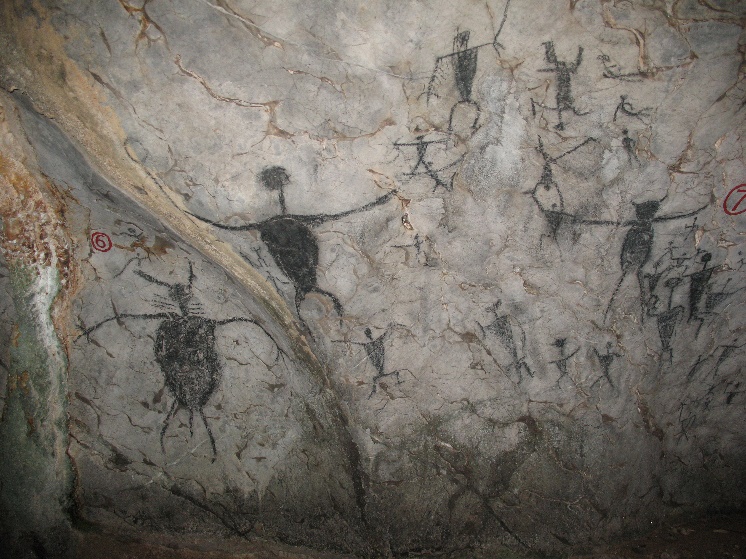


**Fig S2.2**. **Image comparison of panel 6 from 1980 to 2019.** *Left* 1980 photograph showing the application of polyvinyl acetate in toluene (hydrocarbon products) to Panels 6 and 7 (after Solheim 1983:39).; *Right* Panel 6 and the south-eastern portion of Panel 7 in 2019 (photograph Paul S.C. Taçon). Note the darker surface at the bottom and left edge of the panel housing microorganisms (including green microflora, presumably algae and/or lichens) is present in both images.


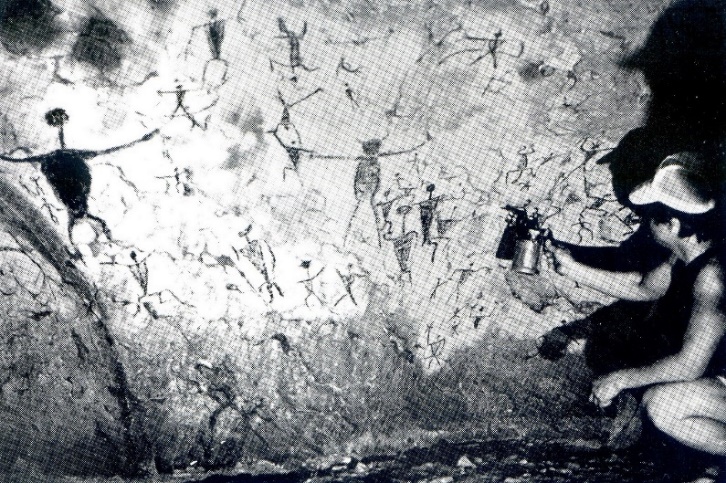

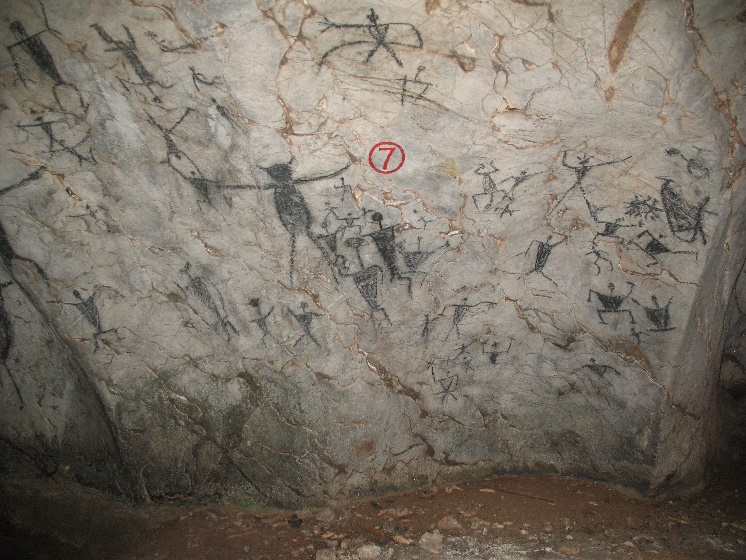


**Fig S2.3**. **Image comparison of panel 6 from 1980 to 2019.** *Left* 1980 photograph showing the application of polyvinyl acetate in toluene (hydrocarbon products), detail of Panel 7 (after Solheim 1983:39).; *Right* Panel 7 and the south-western portion of Panel 6 in 2019 (photograph Paul S.C. Taçon). Again, the darker surface at the bottom of the panel housing microorganisms (including green microflora, presumably algae and/or lichens) is present in both images.


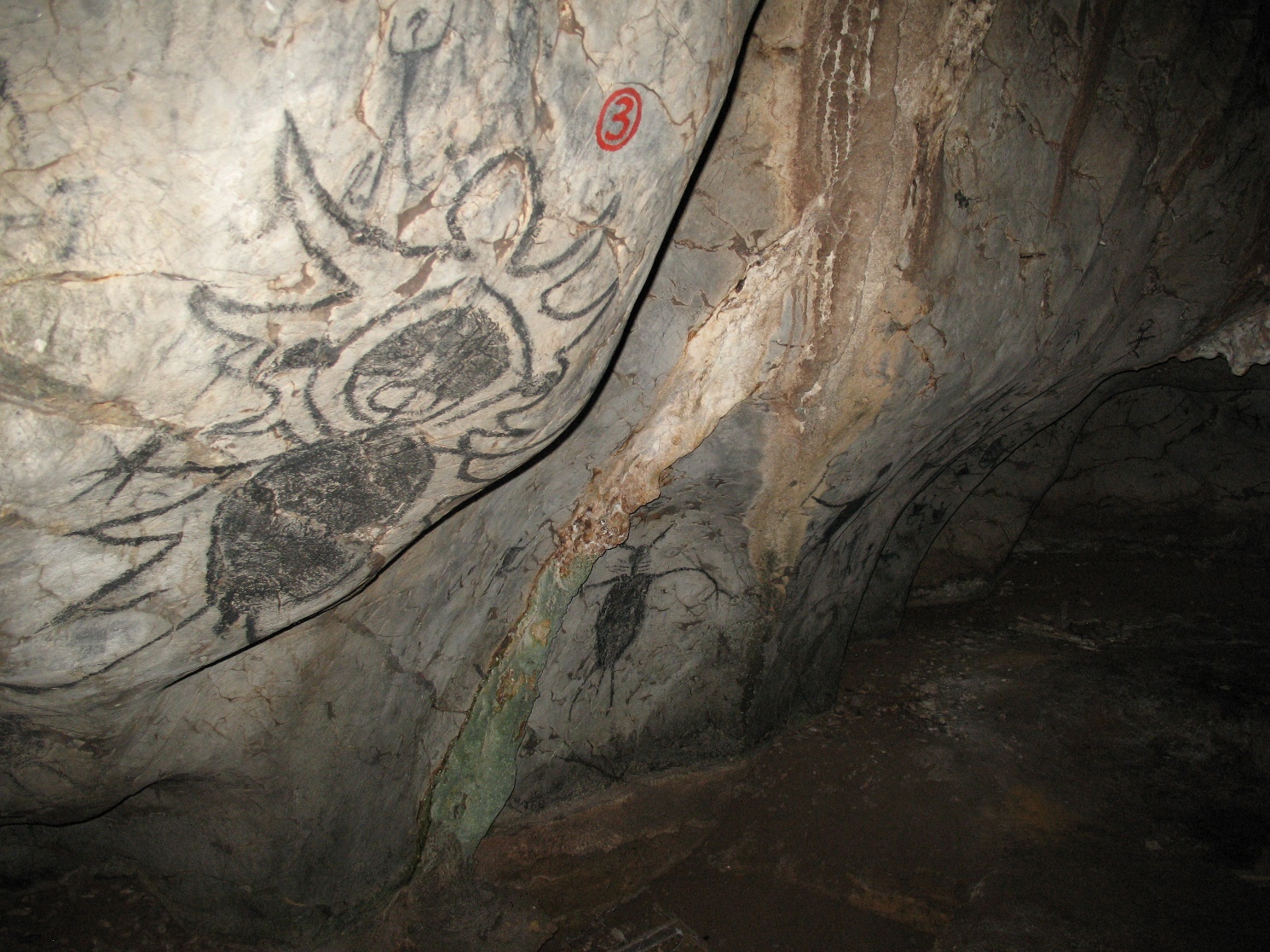


**Fig S2.4**. **Location of dating samples GS2 relative to GS2.** Red arrows indicate the location of dating samples GS3 (foreground) and GS2.
