# Supplementary material for: Rock art and frontier conflict in Southeast Asia: Insights from direct radiocarbon ages for the large human figures of Gua Sireh, Sarawak
Source: PLoS One. 2023 Aug 23;18(8):e0288902. doi: 10.1371/journal.pone.0288902 (PMC10446206; doi:10.1371/journal.pone.0288902)
Supplement: S3 Text — (DOCX) [file pone.0288902.s003.docx]

# Supporting Information

## **S3. Text: ‘Fly through’ 3D model data links, and composite images of the dated anthropomorphs.**

An animated ‘fly through’ of the 3D model generated by Andrea Jalandoni for Gua Sireh can be accessed here:

<https://research-repository.griffith.edu.au/handle/10072/420632>

The DOI for this video is:

<https://doi.org/10.25904/237M-ZW51>

**
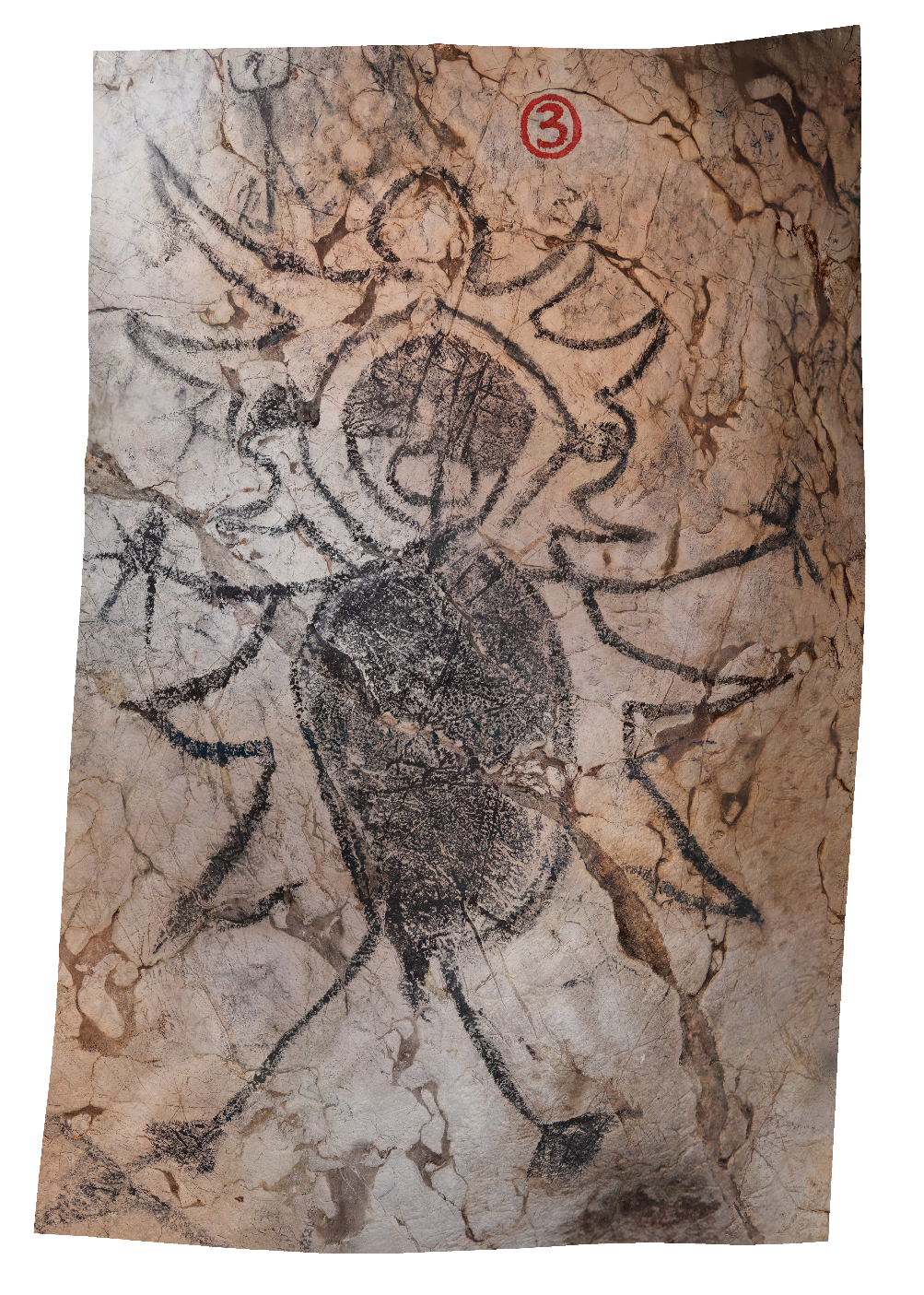
**

**Fig S3.1**. Photomontage of GS3. Red arrows indicate the location of small anthropomorphs on the panel.

**
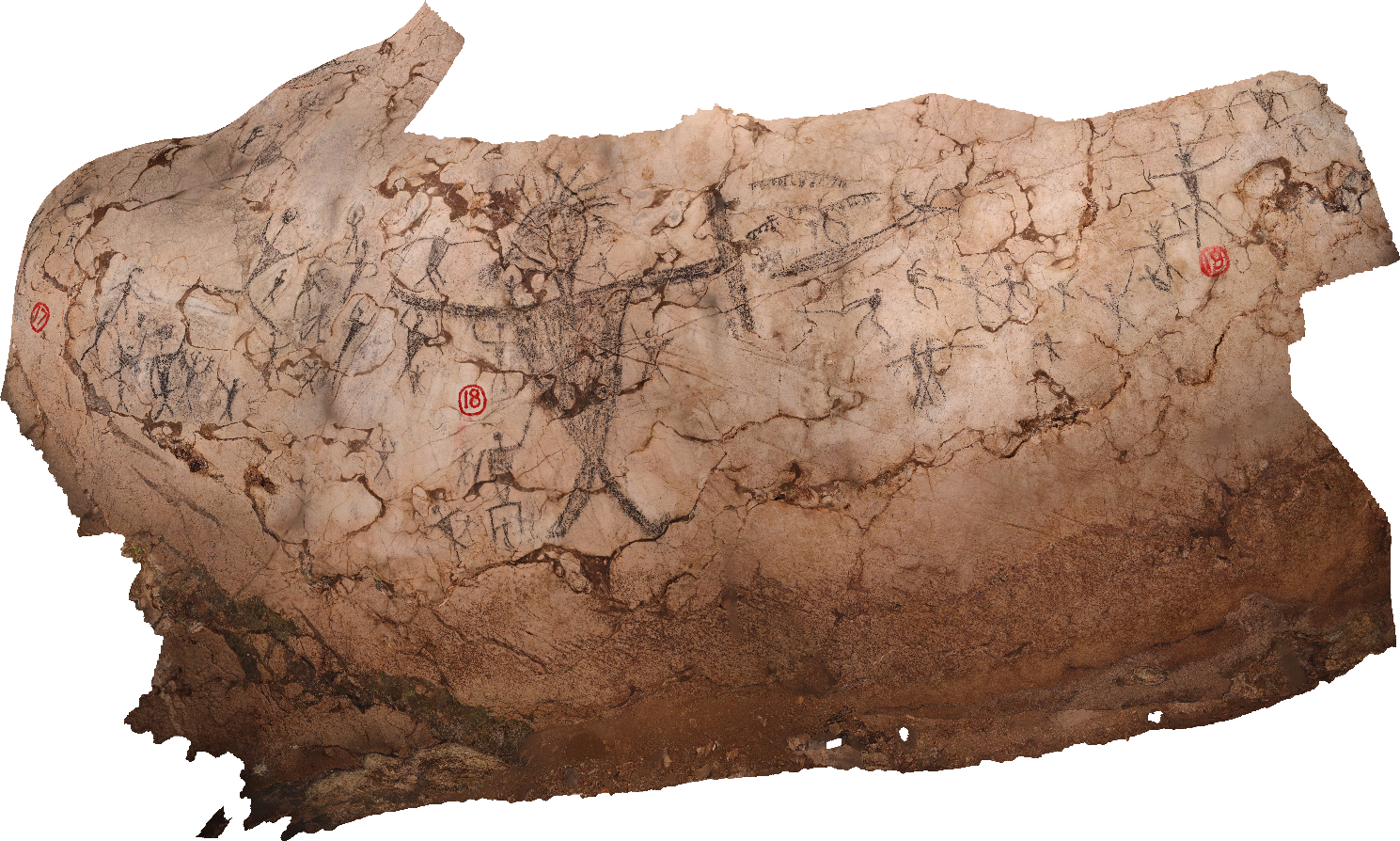
**

**Fig S3.2**. Photomontage of GS4. Overview of panels 17 to 19 showing examples of the scene/groups of figures described by Datan 1990.

**
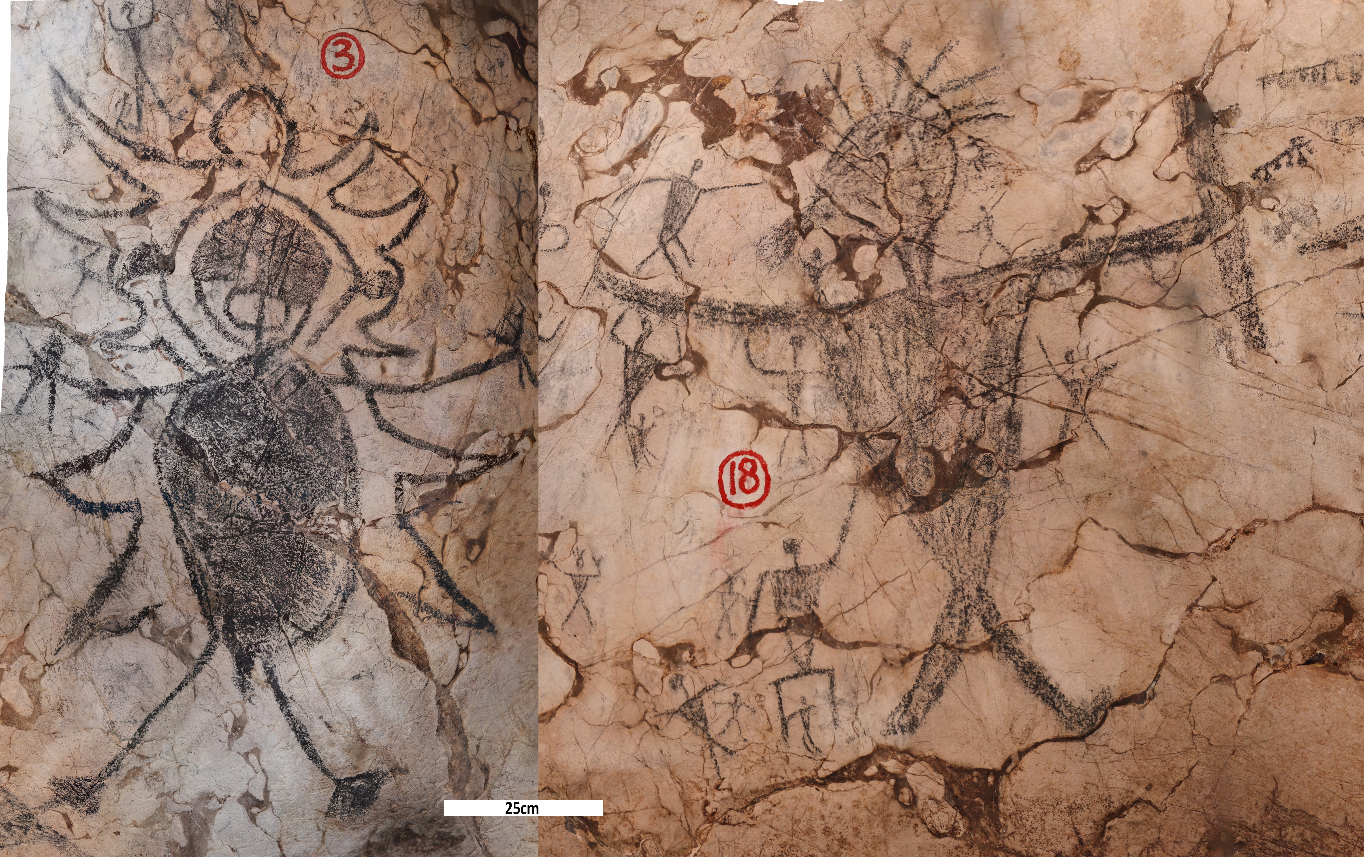
**

**Fig S2.3**. **GS3 and GS4 showing associated small human figures.** Comparative overview of the two large (>75 cm) Gua Sireh human figures dated in this study and the arrangement of small human figure surrounding them (photographed by Andrea Jalandoni 2019).
