# Supplementary material for: Rock art and frontier conflict in Southeast Asia: Insights from direct radiocarbon ages for the large human figures of Gua Sireh, Sarawak
Source: PLoS One. 2023 Aug 23;18(8):e0288902. doi: 10.1371/journal.pone.0288902 (PMC10446206; doi:10.1371/journal.pone.0288902)
Supplement: S4 Text — (DOCX) [file pone.0288902.s004.docx]

# Supporting Information

S4 Text: Scanning Electron Microscope Analyses.

**GS1___________________________________________________**

**



**

**



**

**Fig S4.1**. Comparative view of grain micromorphology in sample GS1.

Overview of grains – dark, larger angular grains with holes along their edge fins are charcoal pigment; lighter coloured ‘fluffy’ grains are limestone; top right image shows sinuous fibres that may contain spider silks, but are more consistent with microflora (the ball shaped end structures) that have been observed in microbial mats on ancient rock art panels in the region (Brumm et al. 2017; Aubert et al. 2018 – bibliographic information in main manuscript).


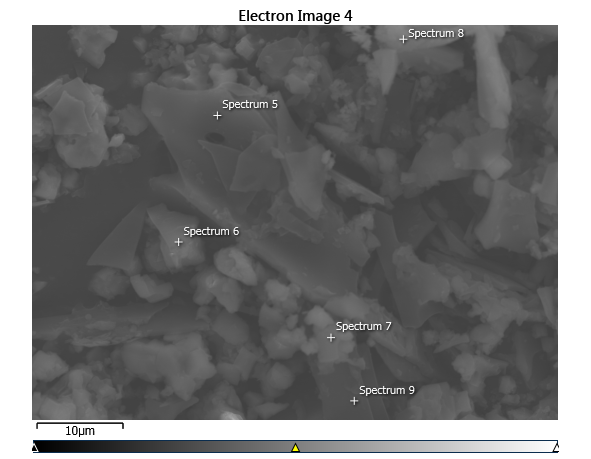

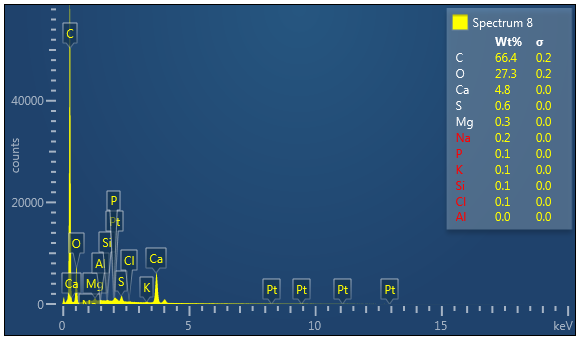

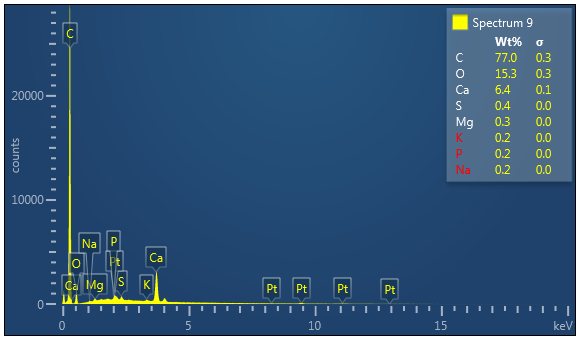


**Fig S4.2**. Grain micromorphology and EDS location with spectra confirming carbon composition of small charcoal grains (Spectrum 8 and 9).















**Fig S4.3**. Details of angular and plate structured dark grains with consistent whole patterns, particularly at their ‘fin’ or edge protrusions, consistent with wood charcoal.


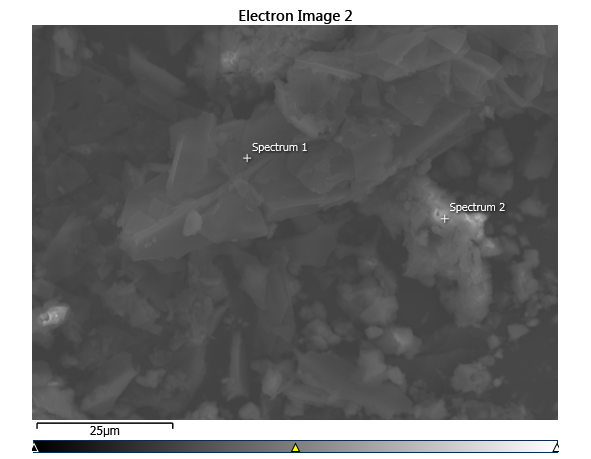

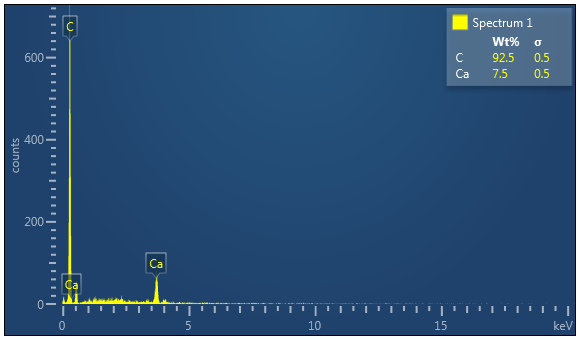

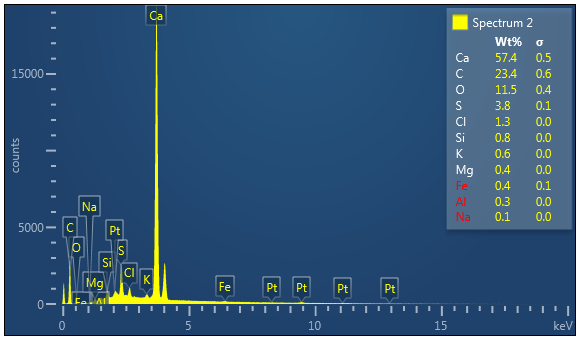


**Fig S4.4**. EDAX spectra of charcoal (spectrum 1) and cave surface (spectrum 2) features.


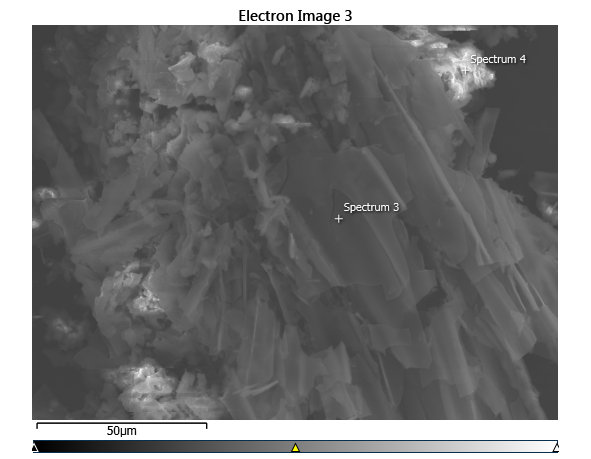

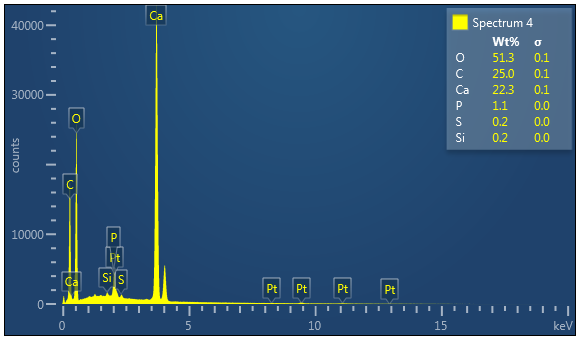


**Fig S4.5**. EDAX of cave surface/weathering feature (spectrum 4) indicating redeposited calcium carbonate and geological weathering (elevated S interpreted as likely gypsum).

**GS2___________________________________________________**

**





**

**Fig S4.6**. Overview of grain morphology (left and centre 10µ, right 1µ scale).

**











**

**Fig S4.7**. Detail of grain structures (top 1µ scale, bottom 10µ).


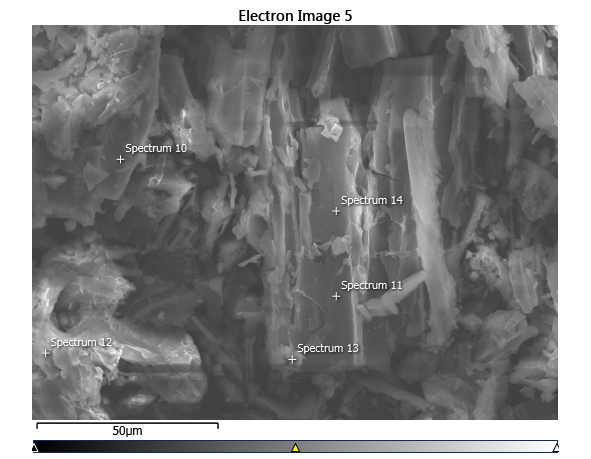

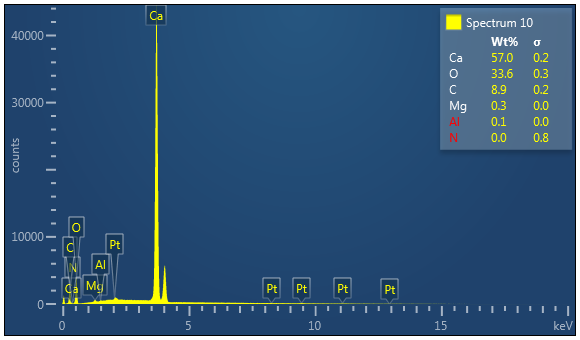

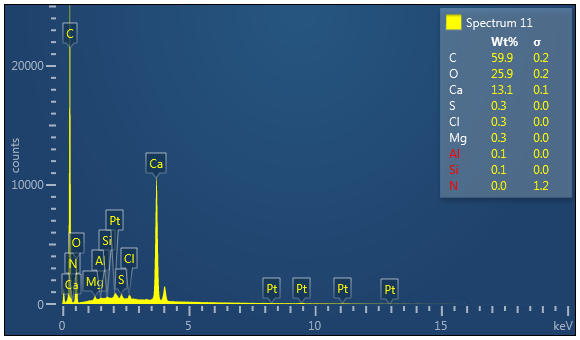

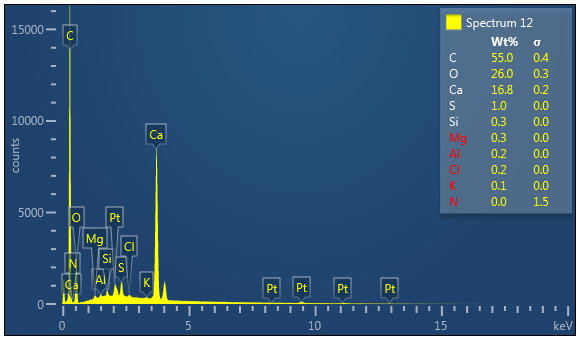

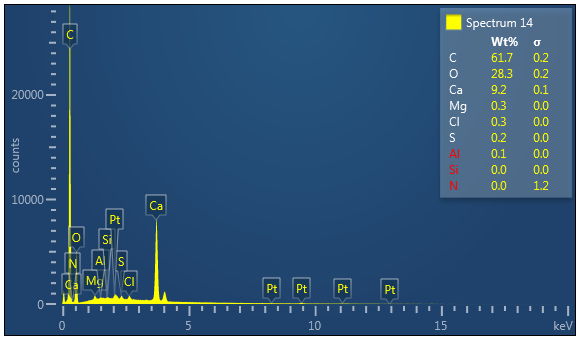


**Fig S4.7**. EDAX chemical profiles of charcoal grain (spectrum 11) and cave surface/weathering detritus (spectra 11, 12, 14).


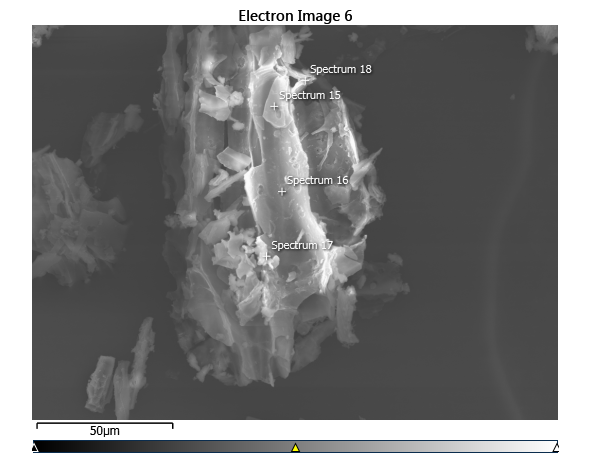

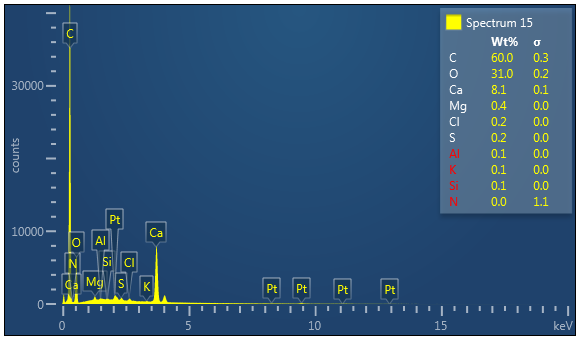

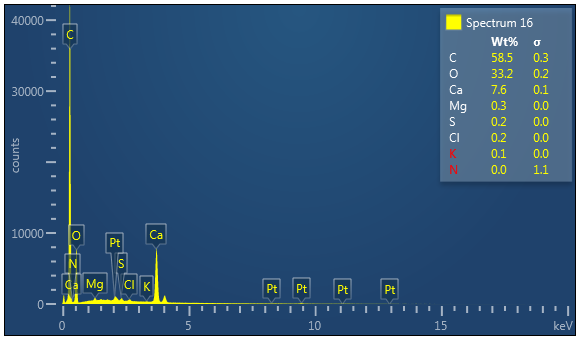

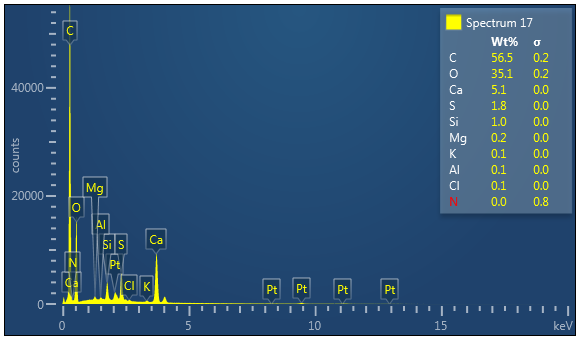

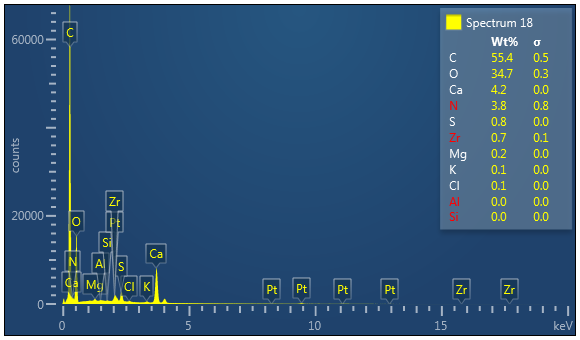


**Fig S4.8**. EDAX chemical profiles of small surface grains stuck to the charcoal pigment.


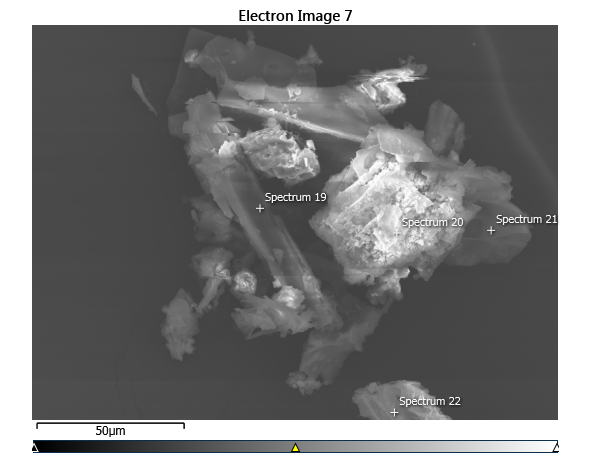

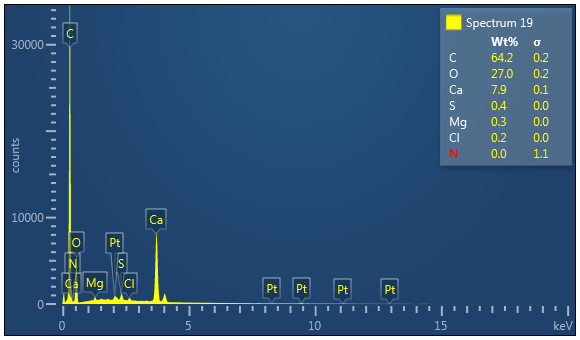


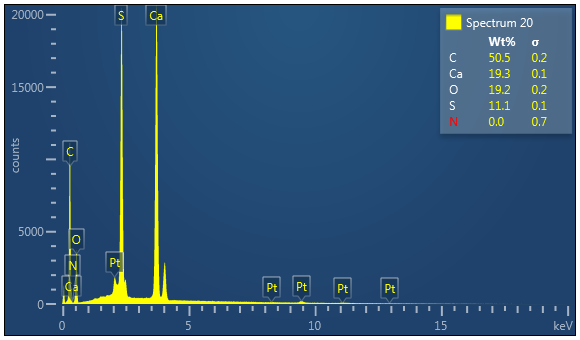

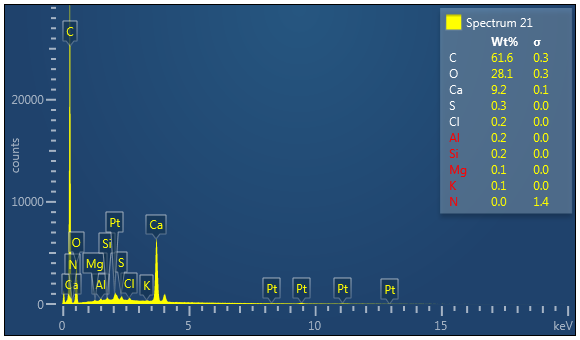

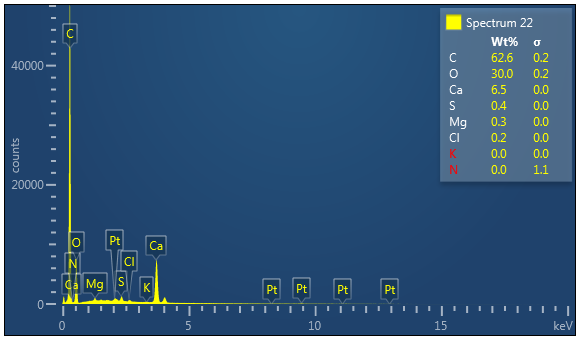


**Fig S4.9**. EDAX chemical profiles of different surface panel constituents including surface geological salts (spectrum 20).

**GS3___________________________________________________**















**Fig S4.10**. Overview of grain structure. Bottom left showing (sparse) microbial activity (10µ scale).







**Fig S4.11**. Detail of the hole structures at the edge/fins and vitriform microbial strands (10µ scale left, 1µ scale right).


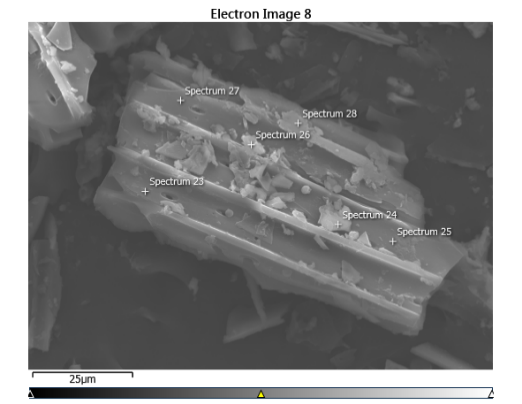

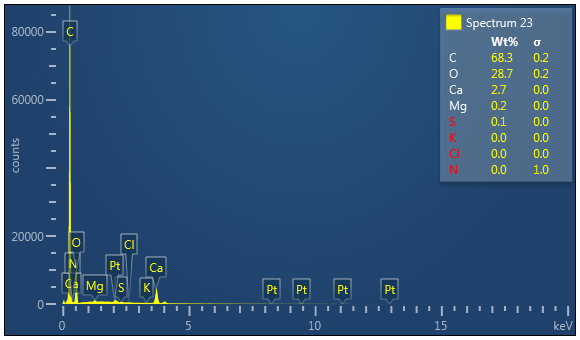

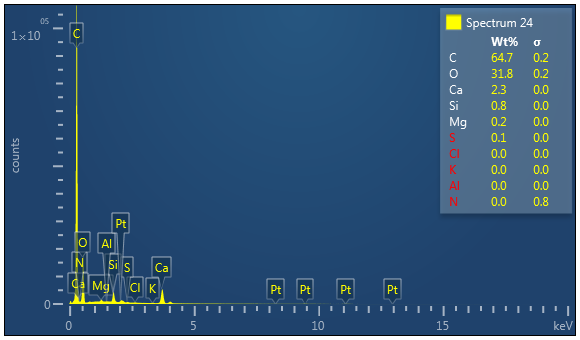

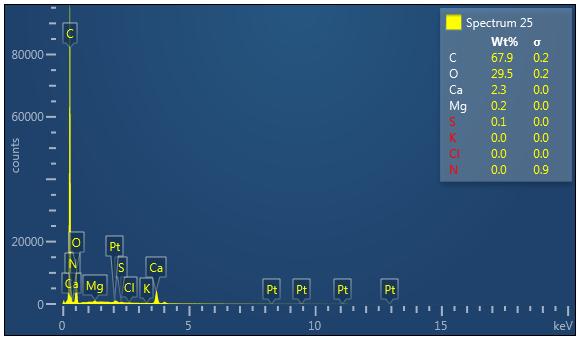

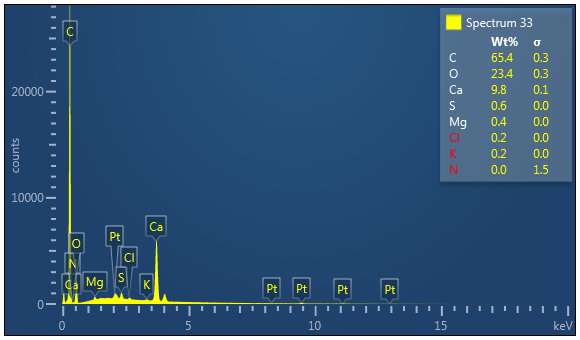

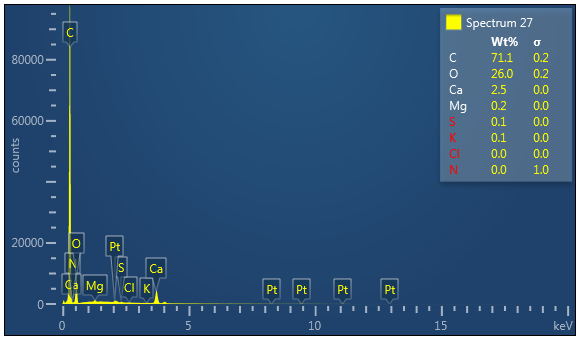

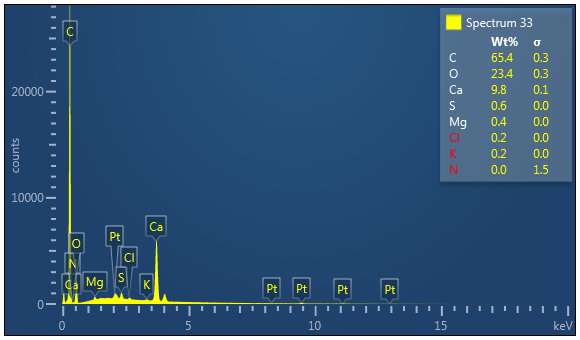


**Fig S4.12**. EDAX chemical profiles of different cave surface constituents from sample GS3.


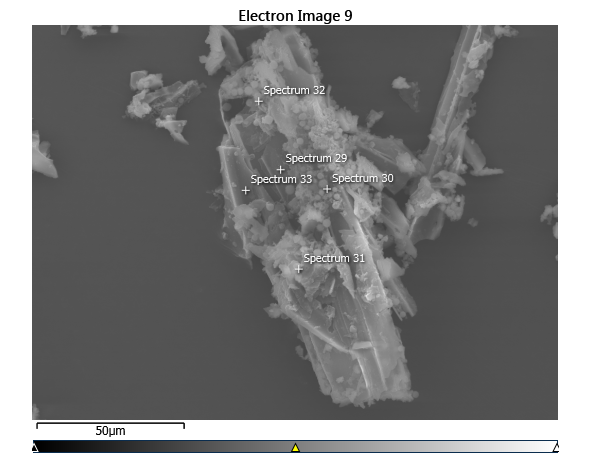

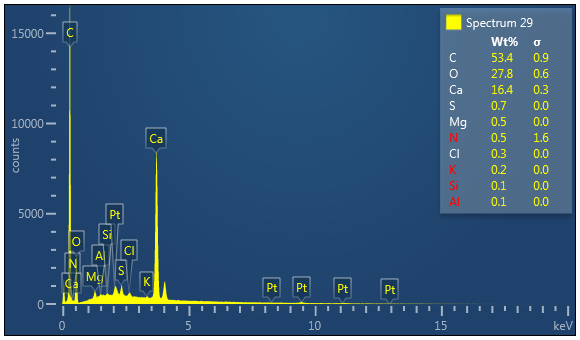

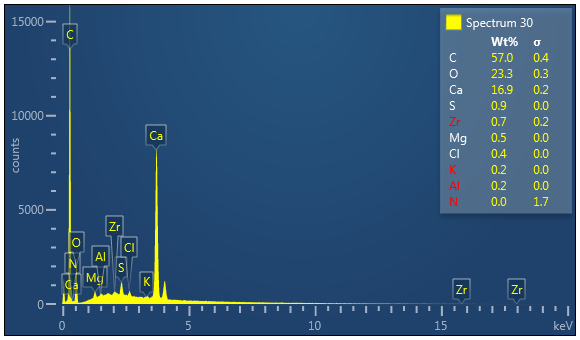

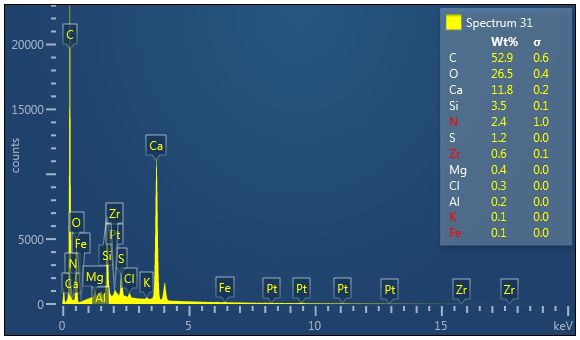

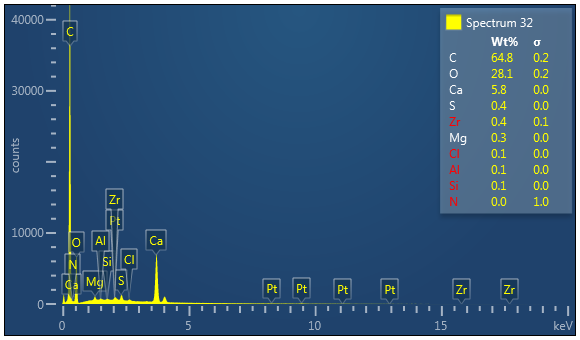

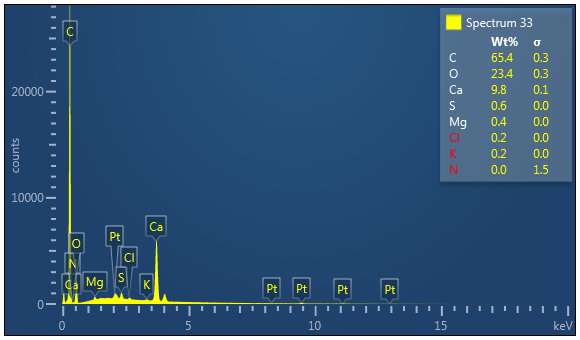


**Fig S4.13**. EDAX chemical profiles of different cave surface constituents from sample GS3.


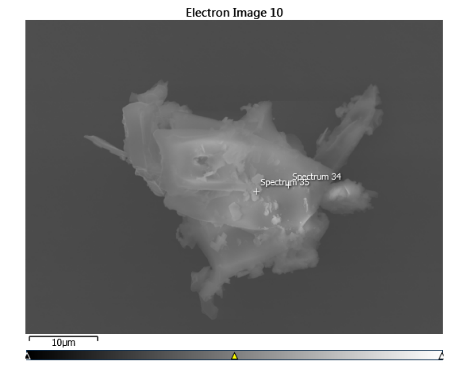

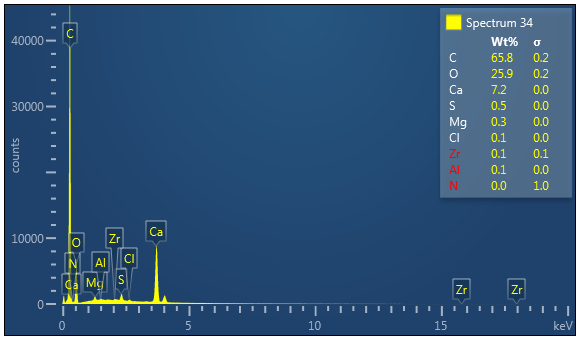

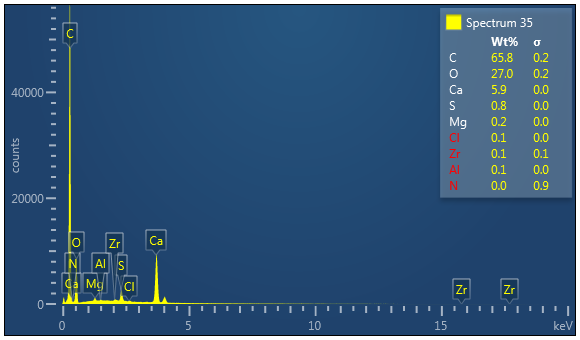


**Fig S4.14**. EDAX chemical profiles of different cave surface constituents from sample GS3.

**GS4___________________________________________________







**

**Fig S4.15**. Detail of grains – this sample has more aggregates and detritus than the other samples (top scale bar 100µ, bottom scale bar 10 µ).















**Fig S4.16**. Detail of the more fragmentary, smaller charcoal grains (in comparison to the other samples). There are still hole structures in the plated fins of the presumable wood charcoal grains (scale bars 10µ).


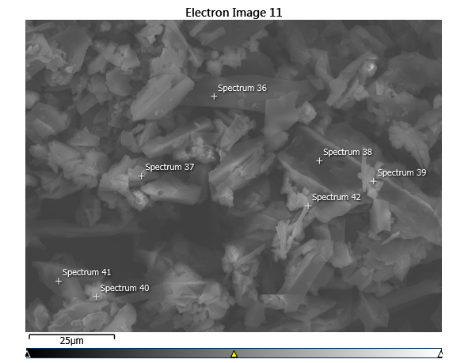

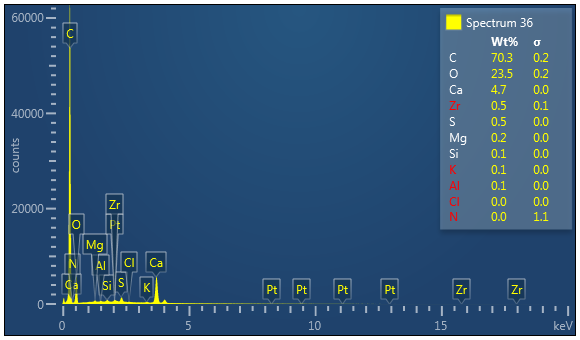

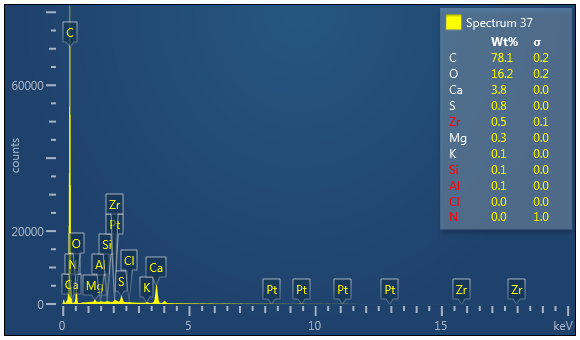

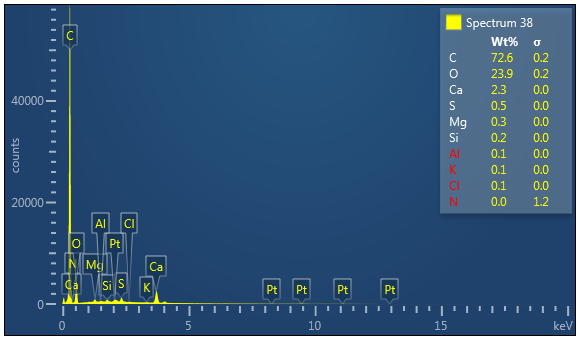

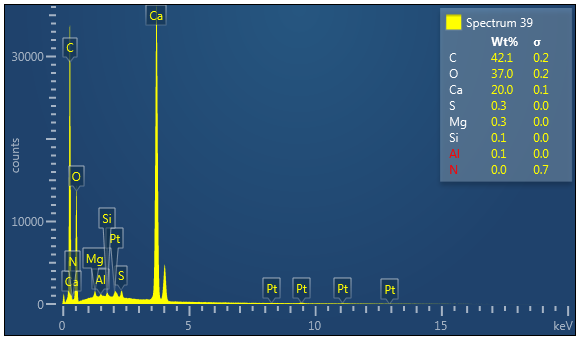

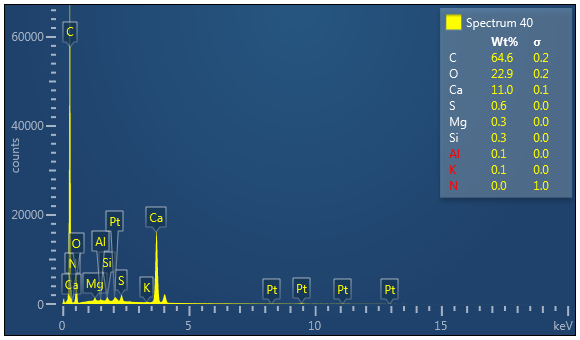

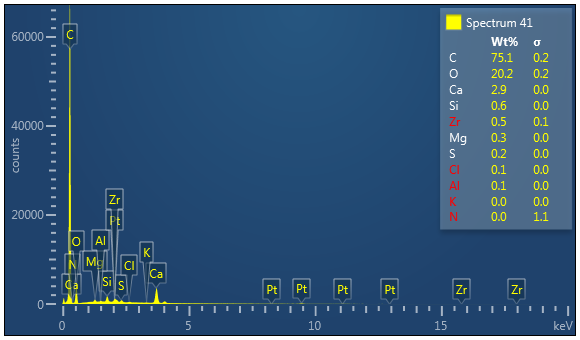

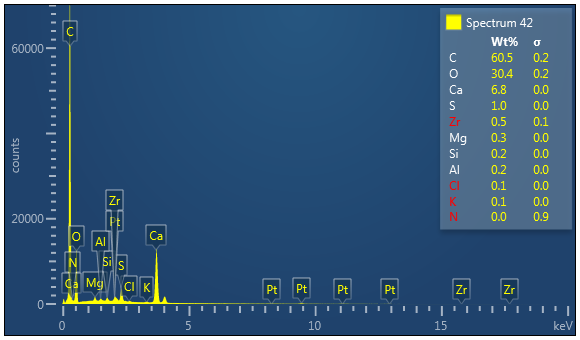


**Fig S4.17**. EDAX chemical profiles of different cave surface constituents from sample GS2.


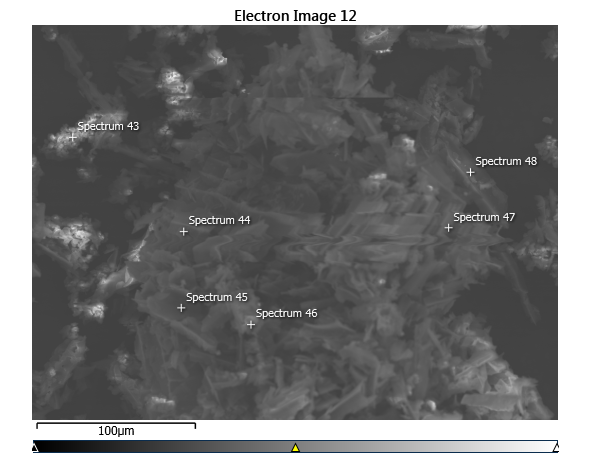

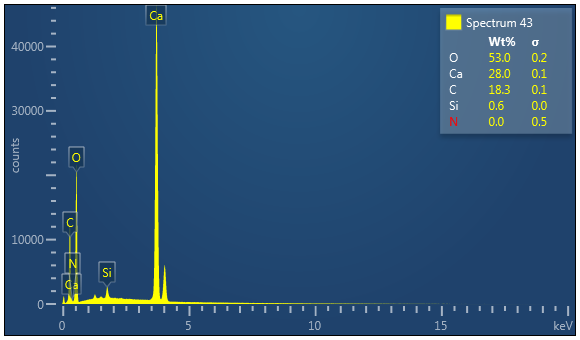

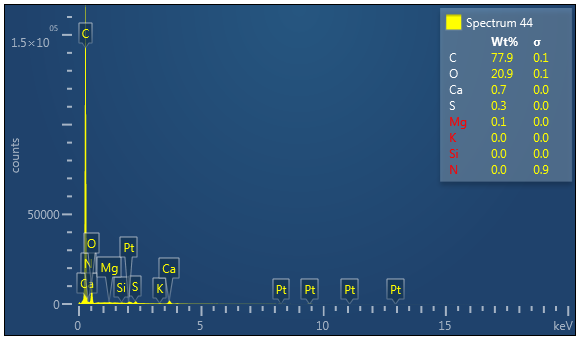

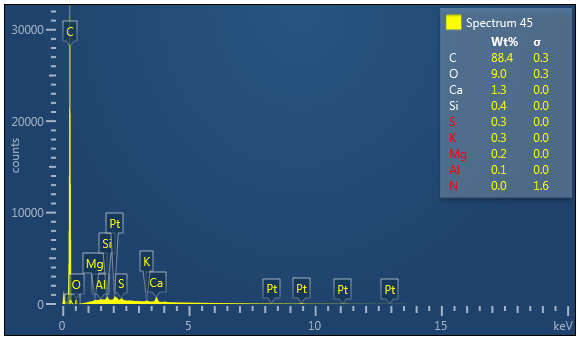

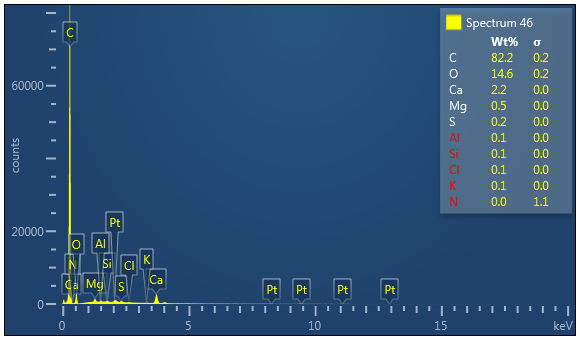

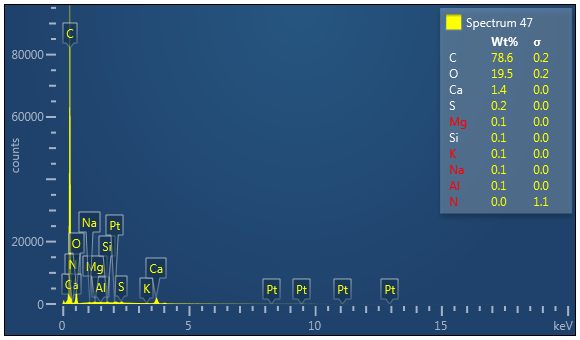

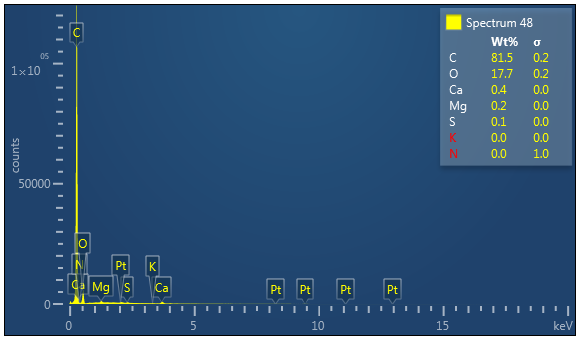


**Fig S4.18**. EDAX chemical profiles of different cave surface constituents from sample GS2.


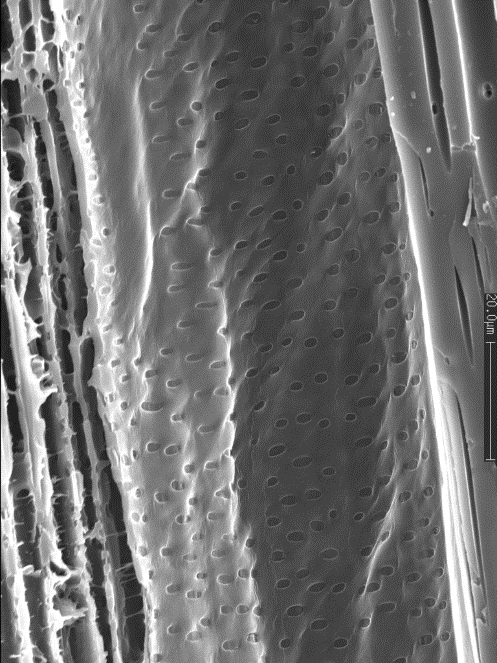




**FigS4.19**. SEM images of reference specimen charcoal from Dendrocalamus giganteus showing opposite to alternate simple pits in metaxylem vessel of vascular bundle (left) and grouped fibres and vessels in a vascular bundle shown in transverse section (right) (image copyright Emilie Dotte-Sarout 2010).
