# Supplementary material for: Rock art and frontier conflict in Southeast Asia: Insights from direct radiocarbon ages for the large human figures of Gua Sireh, Sarawak
Source: PLoS One. 2023 Aug 23;18(8):e0288902. doi: 10.1371/journal.pone.0288902 (PMC10446206; doi:10.1371/journal.pone.0288902)
Supplement: S5 Text — (DOCX) [file pone.0288902.s005.docx]

# Supporting Information

## **S5 Text:** Radiocarbon dating summary.

*Note: no dating target could be produced from sample GS1*

**GS2**

D ^14^C -18.4 2.4 ‰

F ^14^C% 198.2 0.2 %

**Result 149 ± 20 BP**

**Fig S5.1.** Radiocarbon dating results for sample GS2.

**GS3**

D ^14^C -31.7 2.3 ‰

F ^14^C% 196.8 0.2 %

**Result 259 ± 19 BP**

**Fig S5.2.** Radiocarbon dating results for sample GS3.

**GS3**

D ^14^C -16.9 2.3 ‰

F ^14^C% 98.3 0.2 %

**Result 137 ± 19 BP**

**Fig S5.3**. Radiocarbon dating results for sample GS4.
