# Supplementary material for: Rock art and frontier conflict in Southeast Asia: Insights from direct radiocarbon ages for the large human figures of Gua Sireh, Sarawak
Source: PLoS One. 2023 Aug 23;18(8):e0288902. doi: 10.1371/journal.pone.0288902 (PMC10446206; doi:10.1371/journal.pone.0288902)
Supplement: S6 Text — (DOCX) [file pone.0288902.s006.docx]

# Supporting Information

## S6 Text: Example Pandat and Parang weapons from Sarawak and Sabah.


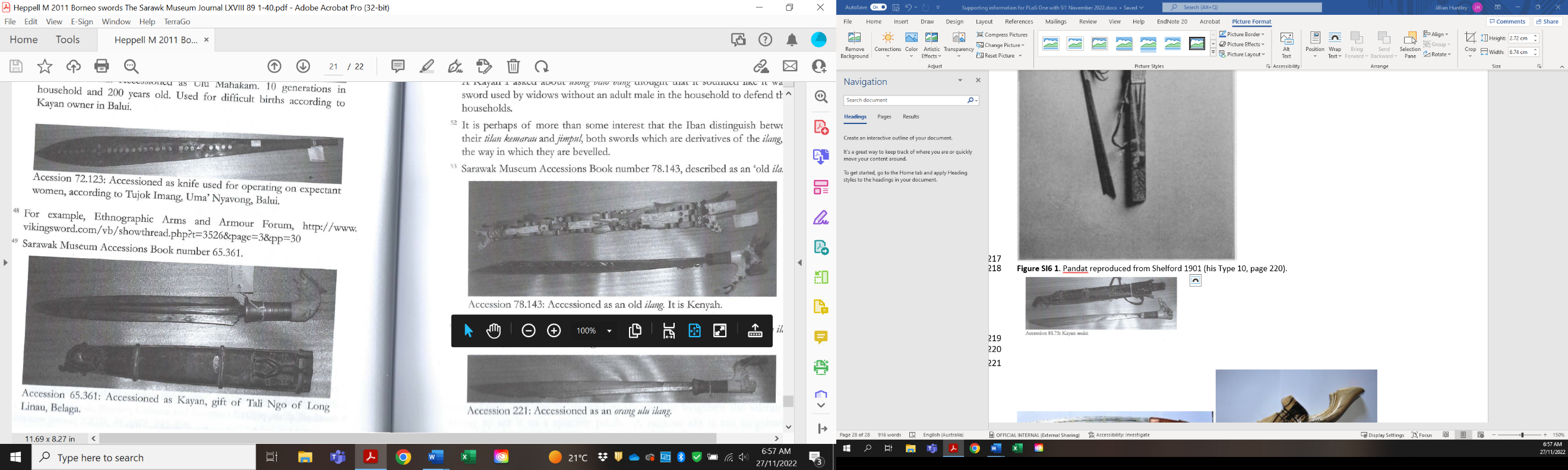

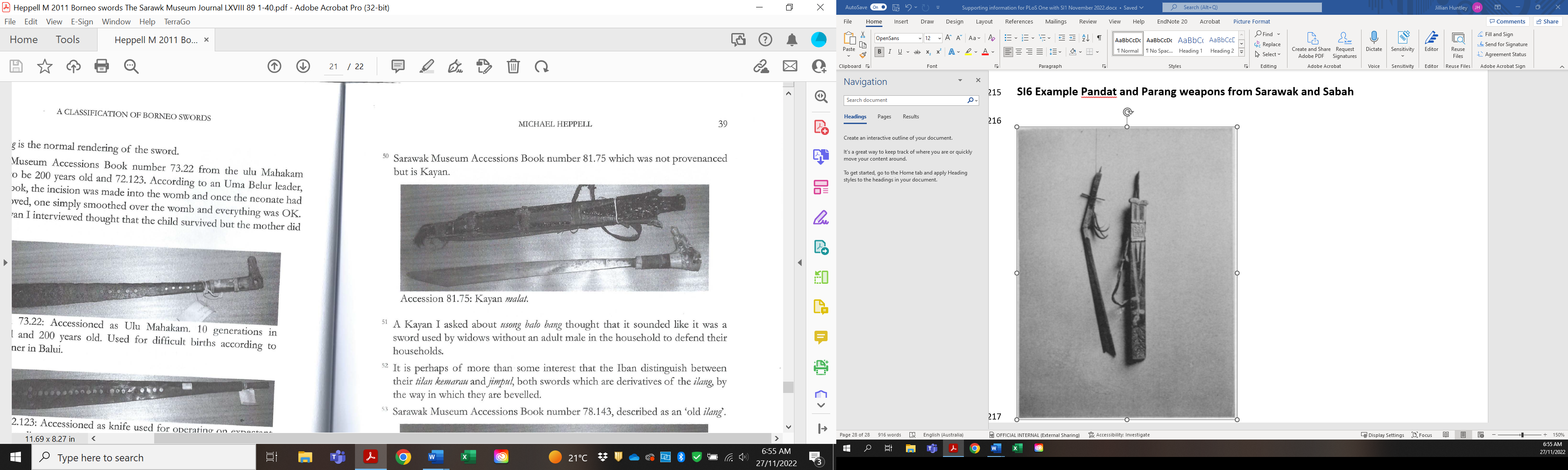


**Figure S6.1.** *Right:* Pandat reproduced from Shelford 1901 (his Type 10, page 220). *Left:* Example Kayan swords held in the Sarawak Museum collection, reproduced from Heppell 2011:39.

**Figure S6.2.** Parang produced by modern master crafters. Left from Sri Aman approxmatley 150 km east of Gua Sireh. Right from Lawas located more than 500 km northerast of Gua Sireh. Reporduced from Mohammed et al. 2018, their Tables 2 and 3.
